# Contact and Non-Contact Ultrasound-Assisted Metal Additive Manufacturing: Recent Progress in Melt-Pool Control

**DOI:** 10.3390/ma19194120

**Published:** 2026-09-26

**Authors:** Nuo Xu, Bo Yuan, Zhong Zheng, Wenting Ouyang, Bowen Gong, Likun Wang, Sainan Ma, Zhanxing Chen, Xinfang Zhang, Qiuwei Xing, Cheng Liu, Xiang Gao

**Affiliations:** 1 School of Materials Science and Engineering, Zhengzhou University of Aeronautics, Zhengzhou 450046, China; xunuo20010910@163.com (N.X.); zxfzua@zua.edu.cn (X.Z.); xqw@zua.edu.cn (Q.X.); 2 Institute of Intelligent Manufacturing Technology, Shenzhen Polytechnic University, Shenzhen 518055, China; yuanbo@szpu.edu.cn; 3 School of Materials and Energy, Foshan University, Foshan 528000, China; zhengzhongmust@163.com; 4 Institute for Composites Science Innovation (InCSI), School of Materials Science and Engineering, Zhejiang University, Hangzhou 310058, China; 12126008@zju.edu.cn (W.O.); 11726046@zju.edu.cn (B.G.); 5 Ningbo Global Innovation Center, Zhejiang University, Ningbo 315100, China; wanglk01@zju.edu.cn (L.W.); sainanma@zju.edu.cn (S.M.)

**Keywords:** metal additive manufacturing, ultrasound assistance, melt-pool control, grain refinement, strength–ductility synergy

## Abstract

Ultrasound-assisted metal additive manufacturing (U-FAAM) has emerged as a promising in situ melt-pool regulation strategy for addressing the challenges of conventional metal additive manufacturing, including process instability, defect formation, and microstructural anisotropy. This review systematically summarizes recent progress in contact and non-contact ultrasound-assisted metal additive manufacturing from the perspective of acoustic energy delivery pathways. The interactions between ultrasonic excitation and melt-pool behavior, thermal transport, solidification-front evolution, defect formation, microstructural transformation, and mechanical performance are comprehensively discussed. Contact ultrasound approaches transmit acoustic energy through solid media. Cavitation can occur only when the melt-side acoustic pressure reaches the required threshold; under such conditions, cavitation-associated effects may act with acoustic streaming and inertial melt flow to enhance mixing, grain refinement, and defect mitigation. Non-contact ultrasound approaches provide improved geometric adaptability and mainly influence melt-pool evolution through acoustic streaming and cyclic pressure fluctuations. The relationships among ultrasonic input conditions, melt-side responses, solidification behavior, and final properties are critically analyzed. Furthermore, current limitations associated with acoustic energy attenuation, coupling efficiency, process scalability, and quantitative characterization of ultrasonic effects within the melt pool are highlighted. This review synthesizes current evidence for ultrasound-driven melt-pool control in the investigated fusion-based AM systems, particularly DED/LDED and WAAM, while recognizing the limited evidence for LPBF.

## 1. Introduction

Metal additive manufacturing (AM) fabricates components by successive cycles of localized melting, rapid solidification, and thermal reheating of previously deposited material [1,2]. Laser powder bed fusion (LPBF) and directed energy deposition (DED) differ in feedstock delivery, heat-source interaction, deposition scale, and process access [3]. Laser DED (LDED), also referred to as laser metal deposition (LMD), uses a laser heat source with wire or powder feedstock [3,4]. Laser engineered net shaping (LENS) is a representative powder-fed LDED process. Wire arc additive manufacturing (WAAM), in contrast, uses an arc heat source and wire feedstock [5]. Despite these distinctions, they are all fundamentally governed by the evolution of the transient melt pool [6,7,8]. Given its central role in governing thermal transport, fluid flow, solidification behavior, and defect evolution, the melt pool has become one of the most intensively investigated thermo-fluid-solidification physical entities in the metal AM field. Its highly transient characteristics directly govern energy utilization, deposition stability, morphology evolution, and defect formation [9]. Consequently, the melt-pool investigation is fundamental to both microstructure regulation and performance optimization. Therefore, various in situ auxiliary-field approaches have been introduced into AM to improve process stability and regulate the resulting microstructure–property relationships.

High-speed X-ray imaging and in situ optical diagnostics have enabled direct visualization of dynamic evolution during metal AM, including keyhole fluctuations [10,11,12], spatter generation [10,11], powder-melt interactions [10,13], bubble migration [14,15], and pore evolution [14,15,16]. Complementary thermal measurements and numerical simulations have further quantified critical solidification parameters, i.e., cooling rates, thermal gradients, and local solidification conditions [13]. These transient processes are strongly coupled through complex thermo-fluid-solidification physical behaviors. However, the resulting microstructure depends strongly on the alloy chemistry and phase constitution. Titanium alloys [17,18,19,20], stainless steels [21], nickel-based superalloys [22,23], and aluminum alloys [24,25] exhibit fundamentally different solidification behaviors due to differences in phase transformation pathways, constitutional undercooling, solute redistribution, and cracking susceptibility. The incorporation of grain refiners and reinforcing particles introduces additional heterogeneous nucleation sites and alters melt-pool transport behavior [26]. These process- and alloy-dependent differences motivate in situ auxiliary techniques that act directly on melt-pool transport and the solidification front. In situ ultrasound is one such approach [27,28,29]. Therefore, a comprehensive understanding of ultrasound-assisted metal AM requires a clear distinction between the acoustic effects introduced by ultrasonic excitation and the subsequent alloy-specific microstructural responses.

Ultrasound field-assisted metal additive manufacturing (U-FAAM) introduces high-frequency mechanical or acoustic energy during the melting and solidification stages. Fundamental studies on ultrasonic melt-processing associate acoustic cavitation, acoustic streaming, cyclic pressure, and inertial forcing with key phenomena, i.e., bubble transport, solute mixing, dendrite loading, fragmentation, and nucleation [27,28,29]. In fusion-based AM, ultrasound has been coupled through the substrate or build platform [30,31], via a local co-moving solid-coupling route near the active pool [32], or through a gas-phase acoustic source directed at the free surface and trailing solidification region [33,34]. Post-deposition ultrasonic treatments (e.g., impact, rolling, or peening) and interlayer treatments can modify the solidified surface; however, they are beyond the scope of this review because they do not directly control the active melt pool or solidification front. Consequently, in-process studies predominantly focus on DED [35,36,37], LMD/LDED [35,36,38], and WAAM [39,40,41], whose open workspaces facilitate energy-source and feed-system integration [37,41,42]. In contrast, LPBF is generally restricted to platform-excitation strategies because of chamber enclosures, powder-spreading constraints, thin layer thicknesses, tight dimensional tolerances, and the risk of powder-bed disturbance [43,44].

Existing reviews summarize basic ultrasonic phenomena or contact-oriented implementations in DED [35,36] and WAAM [36,39]. This review differentiates contact and non-contact ultrasound by tracing how acoustic energy is delivered to the melt pool. Crucially, the nominal equipment settings, i.e., frequency, power, and source amplitude, do not directly equate to the localized pressure, velocity, or vibration fields induced within the liquid. Rather, the net energy input at the melt-pool interface is regulated by a complex interplay of platform clamping, acoustic path length, component modal responses, contact interfaces, gas-phase attenuation, stand-off distance, incidence angle, shielding gas dynamics, and acoustic-field coverage. To establish a unified framework, our comparative analysis adheres to an explicit causal sequence: energy-delivery path, melt-side effective input, dominant physical mechanism, melt pool and solidification-front behavior, defects/microstructure evolution, multilayer printability, and mechanical performance. Build-scale printability is evaluated separately because localized void fractions alone do not establish component-level continuity or the feasibility of macroscopic mechanical testing. Because non-contact ultrasound has so far been demonstrated for a limited range of alloys and process parameters, its broader parametric sensitivity requires further verification.

### Literature Selection and Scope

The literature discussed in this review was collected through searches of Web of Science Core Collection, Scopus, ScienceDirect, and Google Scholar. The search strategy combined metal additive manufacturing-related keywords, including “metal additive manufacturing”, “directed energy deposition”, “laser-directed energy deposition”, “laser metal deposition”, “wire arc additive manufacturing”, “laser powder bed fusion”, and “selective laser melting”, with ultrasound-related keywords such as “ultrasonic vibration”, “ultrasonic-assisted”, “ultrasound-assisted”, “contact ultrasound”, “non-contact ultrasound”, and “acoustic streaming”. The literature search was not restricted by a fixed starting publication year and was updated to August 2026. After duplicate removal, records were screened by title and abstract, followed by full-text screening against the stated eligibility criteria.

Studies were considered when ultrasonic excitation was directly applied during the additive manufacturing process, and its influence on melt-pool behavior, solidification, microstructure evolution, or defect formation was investigated. Studies focusing on post-processing ultrasonic treatment, ultrasonic impact treatment, ultrasonic inspection, or solid-state ultrasonic additive manufacturing were excluded from the main discussion.

## 2. Ultrasound Input Routes

The ultrasound input routes are fundamentally categorized by ultrasonic propagation medium. Contact ultrasound follows a solid path (via the building platform or a local co-moving coupler), whereas non-contact ultrasound crosses a gas-phase gap (i.e., air or shielding gas) to reach the free surface or trailing region of the melt pool. This transmission-medium classification is the organizing framework of this review because each path governs energy dissipation, boundary conditions, and geometric sensitivities. Architectural differences among LPBF [43,45,46], DED [37,47], LMD/LDED [3,37], and WAAM [41,48] further result in different levels of access to the heat source, feedstock delivery system, shielding environment, and component geometry. Open DED [37,42] and WAAM systems [41,48] readily accommodate local probes, vibrating feed paths, or gas-phase emitters. However, such configurations cannot be directly transferred to other AM processes by simply matching the nominal vibration frequency or amplitude. Instead, the net energy input to the melt pool, physical interference with the deposition process, and accessibility of the active melt pool must be reassessed for each process architecture.

### 2.1. Contact Ultrasound Input

Contact ultrasound relies on a mechanically coupled solid transmission path. In platform-based systems, acoustic waves propagate from the transducer, fixture, and substrate through the pre-deposited bulk component to reach the active melt pool [30]. In co-moving local-coupling systems, the acoustic source, or its terminal solid waveguide, translates in tandem with the deposition head, delivering acoustic energy through a contact probe or horn, an adjacent solid or semisolid region, or a vibrating feed wire [32]. While the platform excitation induces a broad acoustic field within the melt pool, the growing component inevitably becomes an active waveguide, introducing height-dependent attenuation and structural resonance effects. Conversely, moving-contact systems dramatically shorten the acoustic path and decouple energy delivery from the total build height, though the effective input remains highly sensitive to the local interfacial dynamics.

#### 2.1.1. Platform-Based Ultrasound Input

A platform-based transducer is mounted to the substrate, build platform, or fixture. Thus, the ultrasonic vibration propagates through the substrate and deposited layers to the evolving melt pool [30,49]. A transducer mounted beneath a DED platform is illustrated in Figure 1a [50]. Su et al. [51] presented a platform-vibration-assisted LDED system in which the amplified ultrasonic vibration was introduced through the substrate beneath the deposited specimen (Figure 1b). A similar representative specimen layout based on substrate transmission has also been reported [52]. These configurations indicate that platform-based systems deliver ultrasonic energy to the melt pool through the substrate and the growing deposit. This mechanically direct transmission path enables straightforward comparison between ultrasound-assisted and conventional processing, making the configuration widely adopted in proof-of-concept studies [22,44,53]. Strong solid-state coupling can transmit ultrasonic energy to both the melt pool and the adjacent solidification front [52,54,55]. When the local acoustic pressure exceeds the cavitation threshold, cavitation may occur in conjunction with acoustic streaming and inertial vibration [56,57,58].

Platform-based excitation has been reported in NiTi alloys [59], nickel-based alloys [22,60], steels and stainless steels [49,53,54,56], titanium alloys [52], and aluminum alloys [24,51]. Local moving-contact configurations are excluded from this platform-based classification [61,62]. Platform-based excitation has been implemented in AM systems, including DED [22,37,49,53,63], LPBF [44], and WAAM [31,56]. Its principal limitation is the sensitivity of ultrasonic transmission to the propagation path. In a contact-based platform system, the ultrasonic energy transmitted to the melt pool generally attenuates with increasing propagation distance. However, wave reflections, structural resonance, and the evolving dynamic characteristics of the growing build produce locally non-monotonic changes in the delivered ultrasonic intensity [50,54,55,56,64]. Near the substrate, the efficient energy transmission may generate acoustic pressures sufficient to induce cavitation. As the build height increases, the transmitted ultrasonic intensity generally decreases. Thus, the acoustic streaming and inertial vibration may become the dominant mechanisms. Ji et al. [65] found that, in the high-intensity solid-transmission comparison, the columnar grains reappeared when the build height reached 10 mm. In addition, it was difficult to produce wall components exceeding 15 mm due to fusion defects developing in the lower region [65]. It is noted that these critical heights are specific to the material system, component geometry, and ultrasonic coupling configuration [65]. Consequently, platform-based excitation must be interpreted together with height-resolved measurements of vibration amplitude, ultrasonic intensity, melt-pool behavior, or microstructural evolution.

#### 2.1.2. Moving-Contact Ultrasound Input

Moving-contact ultrasound systems employ a short solid-transmission path that travels with the deposition region. The ultrasonic energy may be introduced directly through a contact probe or horn near the melt pool [61,62], coupled through the adjacent solidification front or recently solidified material [66,67], or transmitted via a vibration feed wire [32]. A representative local-vibration configuration places the final coupling interface close to the active melt pool (Figure 2a) [32]. Niu et al. [66] put the ultrasonic head behind the deposition location, allowing vibration to propagate through the recently deposited material and act locally on the subsequent solidification region (Figure 2b). A similarly short transmission path also maintains the final coupling interface close to the active melt pool [61]. Hamilton et al. [32] used in situ high-speed melt-pool imaging and directly observed cavitation-driven melt-pool behavior. Yuan et al. [61] combined deposited-microstructure characterization with finite-element acoustic-pressure calculations to support a cavitation contribution; both studies also discussed acoustic streaming and inertial vibration. Compared with platform-based excitation, the principal advantage of these configurations is that the ultrasonic transmission distance is no longer governed by the overall build height.

However, the shortened transmission path shifts the engineering challenge from long-range ultrasonic propagation to maintaining a stable and reproducible local transmission boundary under continuously evolving processing conditions. Effective energy transfer relies on sustained mechanical contact, precise alignment of the coupling element with the deposition region, and accurate synchronization with the moving heat source. Changes in toolpath curvature, surface morphology, layer height, and travel-speed fluctuations continuously alter the local contact geometry, coupling stiffness, and interaction time, thereby influencing the ultrasonic energy transmitted to the melt pool. Moreover, probes operating in close proximity to the melt pool are susceptible to thermal degradation, mechanical wear, and potential contamination of the deposited material. Moving-contact ultrasound has been implemented through a vibrating feed wire [32], a synchronized local contact tool acting on the deposited layer [61,62], and a follow-up ultrasonic head acting through recently deposited material [66]. Feed-wire excitation eliminates the need for a separate contact tool near the melt pool, but it couples wire feeding, ultrasonic vibration, melting, and droplet transfer into a strongly coupled dynamic system [32]. Hence, it imposes more stringent requirements on wire position, tension, phase synchronization, and device integration [32]. Therefore, while moving-contact excitation substantially reduces the influence of overall build height, it transfers the primary engineering challenges to local synchronization, coupling stability, and long-term durability.

Unlike platform-based excitation, the principal source of uncertainty in a moving-contact system lies in the dynamic condition of the local coupling interface. Even under nominally constant ultrasonic operating parameters, the ultrasonic energy delivered to the melt pool may vary with changes in contact force, wire tension, probe position, tool wear, or the phase relationship between ultrasonic vibration and droplet transfer. Moving-contact excitation reduces build-height attenuation, although propagation-path constraints remain. Ultimately, the local coupling interface governs energy input to the melt pool and the potential cavitation response under cavitation-active conditions [62,66].

### 2.2. Non-Contact Ultrasound Input

Non-contact ultrasound eliminates the need for a solid transmission path through the growing component while avoiding direct tool immersion in the melt pool. An acoustic emitter mounted adjacent to the deposition head travels synchronously with the heat source and directs ultrasonic waves through air/shielding-gas toward the free surface and trailing solidification region. Zhang et al. [34] employed a non-contact ultrasound configuration, as shown in Figure 3a,b. Han et al. [68] adopted a similar ultrasonic arrangement. Along this path, acoustic energy experiences attenuation within the gaseous medium, partial reflection at the gas–liquid interface [68], absorption by the melt pool [33,69], and subsequent redistribution by shielding-gas flow and free-surface dynamics [34,68]. The stand-off distance [34,69], incidence angle [68], and acoustic-field coverage [33,68] collectively govern the overlap between the acoustic footprint and the melt pool, particularly the melt-pool tail adjacent to the mushy zone. The numerical simulations identify potential coupling regions [69], whereas the quantitative evaluation of liquid-side acoustic pressure or intensity still depends on experimental measurements or validated indirect calibration [34,68].

A co-moving gas-phase source preserves a nearly constant geometric relationship with the melt pool despite changes in build height and toolpath. This architecture provides excellent geometric compatibility with tall builds [33], curved deposition trajectories [33,68], localized repair operations [34], and manufacturing processes restricting tool intrusion [34]. Geometric adaptability presents the primary advantage of gas-phase ultrasound assistance, whereas acoustic transmission efficiency remains inherently limited. Attenuation within the gaseous medium, together with the substantial acoustic-impedance mismatch at the gas–liquid interface, reduces the energy delivered to the melt pool [33,69]. Meanwhile, the relative source-pool geometry [68], shielding-gas flow conditions [69], and acoustic-field distribution [68,69,70,71] further modulate the applied ultrasonic forcing. A stand-off distance of 35 mm was adopted in the reported 316L deposition configuration [34], whereas Fe-based studies examined the effects of several stand-off distances [69,70,71]; these values remain specific to the corresponding equipment and process geometries. Current evidence indicates a gradual transition from weak acoustic coupling to a measurable melt-pool response as these parameters become progressively optimized, rather than the existence of a universal three-regime operating window. However, within the investigated parameter space, no general instability threshold associated with high ultrasonic power emerged [68].

Although a co-moving source stabilizes nominal distance and incidence angle, the effective acoustic forcing within the melt pool still fluctuates with pool morphology [34], free-surface inclination [34,68], shielding-gas flow [34,69], deposition-path curvature [33], and acoustic-field coverage [68,69]. Hence, practical process control requires simultaneous consideration of the source-pool geometric relationship and experimentally measurable melt-pool responses, since the generator output alone is insufficient to represent the actual acoustic conditions experienced by the liquid metal [68]. Here, we summarize these route-specific differences in Table 1.

## 3. Melt-Pool Evolution in Contact and Non-Contact Systems

The earliest manifestations of ultrasonic excitation appeared in melt-pool geometry, free-surface dynamics, and liquid-metal flow. Concurrent changes frequently occur in particle or droplet entrainment [49,72], bubble transport [34,69], powder capture [49,73], and spatter behavior [31,55]. Collectively, these phenomena modify convective heat transfer and reshape the thermal history surrounding the melt pool [49,55,69,74]. The manner in which these disturbances propagate to the solidification front strongly depends on the ultrasonic transmission route. When the melt-side pressure reaches the required threshold, solid-state coupling can produce cavitation-associated effects [28] together with pressure transients [29], vigorous acoustic streaming [28], and inertial fluid motion [30], thereby altering melt flow and local thermal conditions. By comparison, under the gas-phase operating conditions reported to date, the estimated acoustic intensity within the melt pool remains well below the cavitation threshold for metallic melts. Accordingly, acoustic streaming [68] accompanied by cyclic pressure fluctuations [33] interacting with Marangoni-driven convection represents the most plausible and consistently supported mechanism. The following sections examine this sequence systematically, from melt-pool dynamics to heat transfer and ultimately to the solidification-front evolution. Although thermal conditions exert a strong influence on columnar growth, disruption of epitaxial solidification additionally requires the availability of viable fragments or heterogeneous nuclei, localized remelting, or mechanically induced deformation and transport within the mushy zone [26,29,33].

### 3.1. Melt-Pool Dynamic Response

Contact excitation imposes periodic displacement, acceleration, and pressure fluctuations on the melt pool. Kabir et al. [75] captured melt-pool flow with and without substrate vibration (Figure 4a). The observations showed that ultrasonic excitation introduced additional vortical circulation and enlarged the turbulent-mixing region. The authors attributed the intensified recirculation to acoustic streaming and possible cavitation, which may have enhanced heat and material transport within the melt pool. Yang et al. [55], combining in situ high-speed imaging with high-fidelity multiphysics modeling, showed that substrate-mediated ultrasonic vibration modifies internal recirculation, free-surface flatness, and ripple morphology. In an independent in situ investigation of 316L DED, El-Azab et al. [49] reported that the mean surface residence time of tracked particles decreased from 1.3 ± 0.32 ms to 0.7 ± 0.8 ms under ultrasonic vibration (Figure 4b). This quantitative observation establishes a direct relationship between ultrasonic excitation, particle capture, and the characteristic timescale of material transport. However, the residence-time reduction remains specific to the investigated particle characteristics, entry conditions, melt-pool geometry, and local vibration state.

Contact vibration further changes free-surface oscillation and spatter detachment behavior [31,55,75]. The previous study on WAAM has reported capillary-wave formation, bead widening, and changes in height and penetration [31,65], whereas DED investigations have revealed changes in melt-pool inclination [55,63], powder-stream interaction [73], particle impingement location [49,76], and liquid-metal transport [76,77]. These observations should be interpreted as interconnected manifestations of ultrasonic forcing rather than independent phenomena. Moderate disturbance promotes shorter transport paths [49,76], improved wetting [49,55], and more uniform heat redistribution [55,63]. However, excessive local energy input favors liquid ejection [49,55], reduced powder-capture efficiency [73], pronounced surface rippling [55,75,78], and instability in track overlap [31,65]. A recent DED-LB study on an AISI 316L/CuCr1Zr system similarly showed that ultrasonic excitation promoted finer and more homogeneous phase distributions but also induced pronounced melt-pool perturbations, thereby compromising process stability [79]. Platform-based vibration systems further introduce spatial variability because the transmitted ultrasonic response evolves with increasing build height. By contrast, moving-contact configurations alleviate the transmission-path limitation, although the effective ultrasonic response still depends on coupling stability, source alignment, and the duration of acoustic exposure.

Solid-state transmission affects a large portion of the melt pool and may even extend across the entire pool [30,49,55]. When the transmitted acoustic pressure is sufficiently high, cavitation also becomes possible [30,56]. In contrast, non-contact excitation concentrates acoustic forcing near the exposed free surface and trailing region [34,68]. However, attenuation and imperfect field placement restrict the effective interaction zone [33,68]. Accordingly, contact-based systems more readily approach conditions associated with excessive ultrasonic excitation. In contrast, non-contact systems more frequently experience reduced effectiveness, owing to insufficient energy transfer or suboptimal spatial coupling. Therefore, the process control in configurations using contact ultrasound primarily relies on position-dependent transmission characteristics and local coupling quality. By comparison, non-contact implementations depend on the overlap between the acoustic field and the melt pool together with the resulting melt-side response. Material properties [30], heat-source dimensions [33], melt-pool geometry [49], build position [30,49], local coupling conditions [32], and the delivered acoustic energy [68] collectively determine these distinct distributions of ultrasonic forcing.

Gas-phase ultrasonic excitation initially interacts with the exposed melt-pool surface and the trailing solidification region. Zhang et al. [34] compared time-resolved melt-pool morphologies during conventional LDED and non-contact ultrasound-assisted LDED (Figure 5). After ultrasonic excitation, the melt-pool surface showed more pronounced fluctuations, while the average melt-pool length and width increased slightly, indicating enhanced spreading and wetting of the liquid metal.

Complementary quantitative evidence was reported by Han et al. [33]. At 17.5 W·cm^−2^, non-contact ultrasound maintained a relatively stable melt-pool surface but changed the internal flow from a steady Marangoni-dominated vortex to a high-frequency oscillatory pattern. The maximum flow velocity in the mushy zone increased from 0.726 to 1.678 m·s^−1^. By contrast, the 849.6 W·cm^−2^ high-intensity contact condition produced severe surface depression and ripple formation together with stronger internal melt flow. The higher flow velocity increased the instantaneous hydrodynamic loading on dendrites, while the high-frequency oscillations imposed repeated cyclic loading. These flow characteristics provide the loading conditions considered in the dendrite-deformation and fatigue-fragmentation analysis in Section 3.3.

Zhang et al. [34] further observed plasma deflection and changes in melt-pool dynamics under non-contact ultrasound. Enhanced spreading and wetting promoted the transport of internal bubbles toward the free surface and facilitated their escape. This bubble-escape response is more consistently attributed to ultrasound-induced melt transport, because independent evidence for cavitation was not provided in that study. Incidence angle changes both the location and orientation of the applied acoustic force, thereby shifting the effective interaction region and influencing deposited-track dimensions [68]. Across the studies reported to date, weak acoustic coupling or inadequate overlap between the acoustic footprint and the tail region produces only a limited melt-pool response. By contrast, improved spatial matching modifies melt spreading, wetting behavior, trailing-region flow, and solidification characteristics. Collectively, these observations support a continuous dependence on processing parameters rather than the existence of a discrete three-stage operating window.

### 3.2. Temperature-Field and Heat-Transfer Responses

Ultrasound modifies the temperature field primarily by the redistribution of convective heat transport, although changes in feedstock capture, droplet transfer, free-surface dynamics, and laser absorption may also contribute to the observed thermal response. El-Azab et al. [49] compared the melt-pool temperature profiles and thermal maps with and without contact ultrasound (Figure 6a). The average peak temperature increased from 1550 ± 50 to 1640 ± 90 °C, while the average melt-pool surface area increased from 0.02 ± 0.02 to 0.05 ± 0.04 mm^2^. In a separate substrate-mediated configuration, the width of the monitored high-temperature zone increased from 4.9 to 5.4 mm, the peak temperature within the selected observation region increased from 722 to 784 °C, and the cooling rate decreased from 1207 to 992 °C·s^−1^ [24]. Previous works also introduced expanded hot regions [31], reduced temperature gradients [54], or decreased cooling rates [78]. These observations associate ultrasound-induced changes in melt circulation and surface dynamics with the redistribution of the thermal field.

Non-contact ultrasound introduces substantially less acoustic energy into the melt pool than solid-coupled contact ultrasound because propagation involves both the gas medium and the gas–liquid impedance interface. Thus, the corresponding thermal response is generally weaker and more localized. Zhang et al. [34] compared the thermal cycles and cooling-rate histories of conventional and non-contact ultrasound-assisted LDED (Figure 6b). The reported histories show that the maximum cooling-rate magnitude decreased from approximately 103 to 68 °C·s^−1^ and that the high-temperature interval was prolonged. The authors attributed this localized thermal-history change to modified plasma–melt-pool heat exchange and convective heat transport; it should not be interpreted as a route-independent increase in temperature. A separate Fe-based study combined numerical thermal-field simulation with thermocouple measurements to show that the controlled stand-off distance affected heating and cooling histories at selected locations within the deposit [69]. Both numerical and experimental evidence support this conclusion [69]. Multiphysics simulation is likewise combined with in situ monitoring and post-process characterization in [33], whereas [34,68,70,71] primarily report experimental observations or measurements for their respective configurations. Together, these evidence streams show that thermal or melt-pool responses depend on stand-off distance [69,70,71], incidence angle [68], deposition trajectory [33,34], and acoustic-footprint position [33,68]. This localized thermal response is consistent with the weak and surface-confined excitation produced by gas-phase ultrasound, which interacts primarily with the oscillating free surface and the melt-pool tail.

Temperature gradients, cooling rates, and repeated thermal cycles play important roles in thermal residual stress accumulation during additive manufacturing [80]. Li et al. [69] compared a numerically simulated stress distribution with X-ray residual-stress measurements in non-contact ultrasound-assisted LMD of an Fe-based material system. At a stand-off distance of d = 80 mm, the measured peak tensile stress was 90.5 MPa with a reduction of 45.6%, and the peak compressive stress was 72.6 MPa with a reduction of 25.2%, relative to the ultrasound-free condition [69]. These measurements showed a reduction in residual stress under the investigated processing configuration, although the experimental validation across different systems remains limited. The changes in temperature and cooling-rate characteristics provide qualitative indications of stress evolution, while quantitative reductions require support from direct residual-stress measurements.

Thermal parameters reflect different aspects of solidification. The peak temperature and hot-zone width describe energy redistribution within the melt pool [24,49]. Here, *G* represents the local temperature gradient at the solidification front, *R* is the solid–liquid interface growth rate, *G/R* indicates the columnar-equiaxed growth tendency, *G × R* is the local cooling rate, and *G*^2^*/R* serves as a study-specific indicator. Han et al. [33] extracted *G*^2^*/R* from the calculated thermal field and interpreted it using an extended Hunt solidification map; it was not introduced as a new criterion by this review. When *G* is expressed in K·m^−1^ and R in m·s^−1^, *G*^2^*/R* has units of K^2^·s·m^−3^. This quantity is distinct from *G/R*, which indicates morphological tendency, and from *G × R*, which represents the local cooling rate. Here, *G*^2^*/R* is treated only as the study-specific mapped indicator used by Han et al. [33], not as a universal morphology criterion. The parameters *G*, *R*, and *G/R* characterize local solidification-front conditions [26,74], whereas cooling rate [13,74] and thermal cycling [13,80] influence phase-transformation behavior and stress evolution. In strongly coupled contact ultrasound systems, changes in these thermal parameters interact with cavitation, acoustic streaming, localized remelting, dendrite fragmentation, and fragment transport, collectively disrupting continuous columnar grain development [30,56]. For non-contact ultrasound, *G*^2^*/R* remained above the columnar-growth threshold used by Han et al. [33], even though the solidified microstructure was refined and showed increased equiaxed character. This discrepancy suggests that additional mechanisms, including oscillatory melt flow and acoustic loading of dendrites within the mushy zone, contribute to microstructural refinement beyond the predictions based solely on thermal criteria.

### 3.3. Solidification-Front Disturbance and Dendrite-Fragmentation Mechanisms

Su et al. [51] inferred cavitation- and acoustic-streaming-related grain refinement from post-deposition SEM/EDS/EBSD and property measurements; the study did not directly image cavitation in the melt pool (Figure 7). Cavitation is considered to promote dendrite fragmentation and additional nucleation, while acoustic streaming redistributes the resulting nuclei and homogenizes the melt, thereby weakening directional columnar growth and promoting finer equiaxed grains. Solid-path vibration introduces periodic pressure fluctuations and inertial forces into the liquid melt pool and surrounding semisolid region [81]. Direct synchrotron X-ray studies quantified cavitation bubbles in Al–10Cu melts [57] and visualized bubble dynamics and fragmentation in solidifying Bi–8Zn [58]; these are liquid-metal-processing analogs rather than in situ AM melt-pool observations. Ji et al. [56], combining deposition experiments with CFD pressure-field modeling and analytical assessment, inferred that cavitation and acoustic streaming may coexist when the modeled local pressure exceeds the activation threshold. El-Azab et al. [49] used in situ high-speed video and thermal imaging in DED to measure surface-level melt-pool temperature, geometry, and particle interactions, but not the internal transient pressure field or cavitation bubbles. Taken together, direct analog observations, model-based assessments, and post-solidification characterization converge on cavitation-associated mechanisms under sufficiently strong contact excitation, but their evidence levels are not identical. Equations (1) and (2) relate local liquid-particle displacement to the time-averaged acoustic intensity and peak pressure under the assumption of a locally plane progressive wave.

(1) $$I=\frac{1}{2}\rho c{u_{a}}^{2}=\frac{1}{2}\;\rho c\;{(2\pi \;fA)}^{2}$$

(2) $$P_{a}=\rho cu_{a}=\sqrt{2\rho cI}$$

where *u_a_* is the peak liquid-particle velocity amplitude, *A* is the corresponding peak displacement amplitude, *I* is the time-averaged acoustic intensity, *ρ* is the melt density, *c* is the sound speed in the liquid, *f* is the ultrasonic frequency, and *P_a_* is the peak acoustic-pressure amplitude. Equations (3) and (4) provide a quasistatic estimate of the Blake threshold for an initially spherical gas nucleus [28,29]. These analytical relationships require the acoustic amplitude within the melt rather than the nominal displacement amplitude measured at the external source or horn.

(3) $$R_{B}=R_{0}\sqrt{3+\frac{3R_{0}(p_{0}-p_{v})}{2\gamma }}$$

(4) $$P_{t}=p_{0}-p_{v}+\frac{4\gamma }{3R_{B}}$$

where *R*_0_ is the initial equilibrium radius of the assumed spherical bubble nucleus, *R*_B_ is the Blake critical radius, *p*_0_ is the static liquid pressure, *p_ν_* is the vapor pressure, *γ* is the surface tension, and *P_t_* is the threshold pressure amplitude corresponding to the quasistatic Blake condition. The relations are derived under the assumptions of linear harmonic motion and a locally progressive acoustic wave. The ratio *P_a_*/*P_t_* provides an initial assessment of the required melt-side pressure amplitude by considering the specified nucleus size, material properties, and spatial location. The dynamic cavitation onset is additionally governed by frequency, viscosity, dissolved gas content, nuclei population, acoustic reflection, standing wave formation, structural vibration modes, and free-surface dynamics. These issues influence the actual pressure conditions experienced within the melt.

Under cavitation-active conditions, when the estimated local pressure exceeds the threshold required to activate a pre-existing nucleus, a bubble may grow, oscillate, and eventually collapse [36]. Such collapse can generate intense pressure pulses, microjets, and high local flow velocities [57] and may contribute to dendrite-arm bending, fracture, and fragment transport [56,58]. In moving-contact systems, this high-energy solid transmission path remains close to the melt pool [32]. By contrast, the energy input from platform-based vibration systems exhibits positional dependence. Cavitation-active conditions are more likely near the substrate, while the inferred cavitation contribution diminishes as propagation distance increases and the structural response evolves [30,49,65]. When the local acoustic input exceeds an appropriate level, the accompanying pressure fluctuation and flow disturbance may induce surface ripples, gas re-entrainment, pore formation, and discontinuities in the deposited track [23,49,82].

The non-contact route operates within a different intensity regime. The highest melt-side acoustic intensity reported for non-contact AM reaches 17.5 W·cm^−2^ [33]. By comparison, cavitation during ultrasonic processing of metallic melts is generally associated with acoustic intensities on the order of 100 W·cm^−2^ or even higher [27,28,29]. This reported acoustic intensity remains below the level typically associated with cavitation. Acoustic attenuation further reduces the effective intensity reaching the melt pool. Consistent with the observed flow characteristics, these observations indicate that the reported non-contact systems are governed predominantly by acoustic streaming. The periodic acoustic pressure drives oscillatory streaming, while Marangoni convection continuously redistributes the induced flow throughout the melt pool [33,68].

The oscillatory streaming subjects dendrite arms within the mushy zone to repeated cyclic loading. Equation (5) estimates the bending-stress amplitude of an idealized cylindrical cantilever dendrite arm:

(5) $$\sigma_{b}\;=\;\frac{6\;\mu \;u_{\mathrm{rel}}\;{L_{\mathrm{eff}}}^{2}}{r^{3}}$$

where *σ_b_* denotes the estimated dendrite bending-stress amplitude, *μ* is the melt viscosity, *u_rel_* is the local liquid velocity relative to the dendrite skeleton, *L_eff_* represents the effective unsupported dendrite-arm length, and *r* is the dendrite-arm radius. Equation (6) evaluates the number of loading cycles accumulated during the period of mechanical susceptibility:

(6) $$N_{\mathrm{eff}}\;=\;f{\;t}_{\mathrm{eff}}$$

where *N_eff_* denotes the actual number of ultrasonic loading cycles experienced by the dendrite, *f* is the ultrasonic frequency, and *t_eff_* represents the duration over which the selected dendrite geometry remains within the mechanically susceptible mushy zone interval while being subjected to the modeled oscillatory flow. Equation (7) applies a Basquin-type relationship to describe the corresponding fatigue response [33]. This framework correlates the local relative velocity between the liquid and dendrite, dendrite-arm geometry, exposure duration, and the high-temperature fatigue properties. For ultrasonic excitation at 20 kHz with an effective loading duration of approximately 0.134 s, the accumulated cyclic loading reaches about 2680 cycles.

(7) $$\sigma_{a}=\sigma_{f}^{\prime }{\;(2N_{f})}^{b}$$

where *σ_a_* is the alternating stress amplitude, *σ_f_*′ is the fatigue-strength coefficient, *N_f_* is the model-predicted number of loading cycles to fatigue failure, and *b* is the fatigue-strength exponent. Under the assumption of fully reversed loading, *σ_a_* ≈ *σ_b_*, and the fracture criterion is simplified to *N_eff_* ≥ *N_f_*. The reported condition of *f* = 20 kHz and *t_eff_* ≈ 0.134 s corresponds to *N_eff_* ≈ 2680 cycles [33]. For the selected slender dendrite geometries, *N_eff_* ≥ *N_f_*, indicating that the model-based fatigue-fracture criterion is satisfied under the adopted geometry, loading, and fatigue-property assumptions.

The estimated melt-side acoustic intensity remained below the cavitation threshold, while the calculated flow oscillated across the melt-pool tail and mushy zone. Han et al. reported that the predicted bending stress and fatigue life satisfied the adopted model criterion, while the solidified microstructure showed disrupted columnar growth with an increased fraction of equiaxed grains. Together, these results provide a model-supported interpretation consistent with a possible pathway involving acoustic-streaming-induced cyclic dendrite loading, fatigue fragmentation, fragment transport, and suppression of columnar growth [33]. However, direct experimental evidence of fragmentation occurring in the liquid state remains unavailable. High-spatiotemporal-resolution in situ methods are therefore still required to observe the internal melt-pool flow field and any liquid-state fragmentation directly.

Fragments that may be produced by repeated bending, fatigue fracture, or local remelting are considered to be transported by acoustic streaming [28,29]. Those that survive in the undercooled liquid may interrupt epitaxial growth and act as nuclei for grains with different crystallographic orientations [33]. Figure 8 presents this proposed sequence, in which gas-phase acoustic forcing couples with Marangoni convection to sustain oscillatory streaming across the melt-pool tail and mushy zone, providing a possible fatigue-fragmentation pathway followed by fragment transport and fine equiaxed-grain formation. Zhou et al. [83] summarized non-contact ultrasound input, melt-pool flow, and recrystallization-related grain-boundary evolution in deposited alloy steel. The fractions of high-angle grain boundaries (HAGBs) and low-angle grain boundaries (LAGBs), dislocation-density distribution (DLD), kernel average misorientation (KAM), and local recrystallization features characterize the grain-boundary, dislocation structure, and local crystallographic orientation after solidification. These microstructural characteristics present the cumulative response of the solidified material to the preceding flow and mechanical loading [83].

The dominant mechanisms and their effective ranges depend on both the ultrasonic transmission route and the build location. A moving-contact system maintains a short solid transmission path with high acoustic input, potentially allowing cavitation-associated effects [32] to coexist with acoustic streaming under cavitation-active conditions. A platform-based contact system may achieve similar conditions in regions close to the substrate [30,49,65]. However, with the propagation distance increasing, reflection, resonance, and structural vibration modes alter the acoustic pressure reaching the upper melt pools [30,49,50,65]. A non-contact system relies primarily on oscillatory streaming [33,68], cyclic acoustic pressure [68], and their interaction with Marangoni convection [33]. Although the underlying mechanisms differ, all three transmission routes have the potential to disrupt columnar growth within the effective range defined by their respective acoustic transmission characteristics [30,32,33].

## 4. Defect Control and Solidification-Microstructure Evolution

Responses of the melt pool and solidification front are retained in the deposited material as defects, grain morphology, crystallographic orientation, solute segregation, and second-phase distributions. These microstructural features preserve the signature of the applied acoustic input. Their engineering significance ultimately depends on whether the corresponding processing conditions also enable stable multilayer fabrication and reproducible mechanical-property evaluation [33,49,68].

### 4.1. Densification and Defect Suppression

Porosity, shrinkage, lack of fusion, and cracking originate through distinct formation mechanisms. Gas porosity is governed by bubble entrapment, migration, and escape [14,15]. Keyhole porosity results from keyhole instability and collapse [10,84]. Shrinkage porosity is controlled by liquid feeding during the final stage of solidification [6]. Lack of fusion arises from insufficient penetration, wetting, or track overlap [5,85]. Cracking is a recurrent defect in metal AM [9]. Additional defect pathways are associated with powder oxidation [86], energy input [87], vapor-driven melt redistribution [88], and powder-melt interaction [72]. Ultrasound influences these defects through melt transport, free-surface stability, liquid feeding, and thermal-stress evolution. Changes in relative density alone are insufficient to identify the governing mechanism.

Su et al. [51] compared defects of AM-ed AA7075 with and without ultrasound assistance, and found that contact ultrasound markedly reduced visible pores and shrinkage cavities (Figure 9a). This implies that the induced ultrasound improves melt transport and liquid feeding during solidification. X-ray computed tomography (XCT) of ultrasound-assisted AZ31 DED revealed volume-porosity reductions of about 81% and 90% at ultrasonic powers of 60 and 90 W, respectively [89]. Interlayer lack-of-fusion defects were replaced by smaller and more uniformly distributed pores [89]. Ultrasonic-frequency pulsed-arc WAAM of 2024 Al reduced porosity while refining the solidification microstructure [90]. In AlMgScZr alloys, both porosity and micro-shrinkage were suppressed, although residual fusion-line defects remained [24]. Peng et al. [60] showed that large pores were present at 12 kHz, whereas they were markedly suppressed at 20 kHz (Figure 9b). At 16 kHz, large pores of approximately 100 μm were still present, whereas the deposit porosity decreased to 0.08% at 20 kHz [60]. Contact ultrasound studies indicate the presence of a bounded processing window. A moving-contact system shortens the acoustic transmission path. Local collapse of acoustic coupling or track discontinuity may still occur because of coupling loss, probe position, droplet disturbance, probe wear, or contamination [32]. In situ observations further demonstrate that ultrasonic vibration alters pore trajectories. The beneficial effect declines when the transmitted vibration weakens or destabilizes the melt pool [49,82].

In another study, non-contact ultrasound at 17.5 W·cm^−2^ slightly reduced the total pore volume and produced a more dispersed three-dimensional pore distribution in Inconel 718 [33]. In a separate contact-ultrasound study of Inconel 718, porosity decreased at one vibration frequency but increased again at a higher frequency, accompanied by an increase in maximum pore size [23]. In ultrasound-assisted LMD of 316L stainless steel, Rabiey et al. reported reductions in pores and microcracks compared with the condition without ultrasonic assistance [91]. For 316L stainless steel, fewer pores were observed in the deposited cross-section under non-contact ultrasound (Figure 9c) [34]. For 316L stainless steel, improved spreading and wetting promoted the migration of internal bubbles toward the free surface, reducing the pore population in the deposited cross-section [34]. The improvement was attributed to acoustic-streaming-assisted bubble migration and escape. For non-contact systems, defect mitigation depends on whether sufficient acoustic energy reaches the melt-pool tail and the late-solidifying liquid. The stand-off distance affects deposition depth [69], thermal history [69], pore formation [69], and cracking [71] in Fe-based deposits. The densification response requires sufficient field overlap with the late-solidifying region, and the incomplete overlap leaves residual shrinkage and crack-initiation pathways [69]. When the transmitted acoustic field is weak, coverage is incomplete, or the acoustic footprint fails to overlap the melt-pool tail, the dominant defect formation mechanisms remain largely unchanged. Within the reported ranges of ultrasonic power and incidence angle, continuous deposition tracks were maintained, and the defect evolution was governed mainly by coupling efficiency and field placement rather than excessive melt-pool disturbance.

### 4.2. Grain Refinement and Columnar-to-Equiaxed Transition

Grain refinement [26], geometric equiaxialization [29], and the columnar-to-equiaxed transition (CET) [26,29] describe different aspects of microstructural evolution. Grain refinement refers to a reduction in the mean grain size. Geometric equiaxialization is characterized by a lower grain aspect ratio or a shorter columnar length, indicating weaker directional growth. CET is achieved when trans-layer columnar grains are consistently replaced by a predominantly equiaxed grain structure. Therefore, these metrics characterize microstructural scale, grain morphology, and growth continuity, respectively [26,29].

Figure 10 distinguishes grain-size refinement from changes in growth morphology. Peng et al. [60] showed progressive refinement of the γ grains on the X–Z plane as the contact ultrasound frequency increased from 12 to 20 kHz (Figure 10a). At 20 kHz, the microstructure showed markedly finer and more equiaxed grains than those observed at the lower frequencies. Increasing contact ultrasound frequency also refined the dendritic or cellular structure in Inconel 718 [23]. Hamilton et al. [32] showed that moving-contact excitation at 20 kHz reduced the mean grain size from 164 ± 39 to 52 ± 2 μm relative to the 0 Hz condition (Figure 10b). The columnar grains extending up to approximately 500 μm without vibration were substantially shortened and refined at 20 kHz, indicating weakened directional grain growth. Ultrasonic LMD of 316L similarly replaced aligned columnar dendrites with equiaxed grains [92]. Zhang et al. [34] reported that non-contact ultrasound reduced the average grain size of 316L stainless steel from 68.8 to 48.9 μm (Figure 10c). In another 316L study, the average grain size decreased from approximately 98 to 34 μm, while the equiaxed grain fraction increased from about 28% to 92% under non-contact ultrasound [68]. In ER70S-6 wire-arc DED, an ultrasonic amplitude of 15 μm produced a refined region covering 94.6% of the cross-section [93]. In H13 tool steel, Masaylo et al. reported that 28 kHz ultrasound-assisted L-DED produced a finer and more equiaxed microstructure [94]. In Fe-based deposits, both grain size and grain morphology changed with stand-off distance and sampling height [69]. Height-resolved electron backscatter diffraction (EBSD) maps further showed that the microstructural response varied with sampling position [24]. Grain size and equiaxed fraction quantify microstructural scale and morphology, whereas confirmation of CET requires the disappearance of trans-layer columnar continuity.

Grain refinement can develop into a change in growth morphology. Zhang et al. [50] applied a contact ultrasound-assisted WAAM method to fabricate NiTi alloy. The average grain size decreased from 147.3 to 103.3 and then 72.2 μm, with the ultrasonic intensity increasing, and the mean grain aspect ratio decreased from 4.1 to 3.6 and finally 1.8 (Figure 11a). The equiaxed grain fraction increased from 28.9% to 41.3% and then 87.4%. The intermediate condition still contained elongated grains. Under the highest ultrasonic intensity, the aspect ratio fell below the study’s criterion of 2.5, and the equiaxed grain fraction reached 87.4%, indicating a nearly complete CET. High-intensity contact ultrasound also transformed columnar prior-β grains in Ti6Al4V into fine equiaxed prior-β grains with an average size of approximately 100 μm [30]. Similar responses were reported in ER321 stainless steel [95] and Mg-Al alloys [81], where synchronous ultrasonic excitation weakened columnar inheritance and increased equiaxed grain populations. An Al-Cu study further demonstrated that the degree of grain refinement differed between regions receiving different levels of effective acoustic inputs [96]. In platform-contact systems, the microstructural response also varies with build height. The possible contributions of cavitation-associated effects, acoustic streaming, dendrite fragmentation, and fragment transport are expected to vary as the acoustic transmission path changes [33].

Non-contact ultrasound influences the solidification region primarily through acoustic streaming rather than cavitation. At a melt-side acoustic intensity of 17.5 W·cm^−2^, coarse columnar grains were replaced by a much finer and more equiaxed microstructure under non-contact ultrasound [33]. A separate study on 316L stainless steel reported a similar evolution, with the equiaxed grain fraction reaching 92% under the strongest 30° incidence condition [68]. The corresponding thermal analysis showed that the thermal condition represented by *G*^2^*/R* remained within the study-defined columnar-growth region, implying that thermal redistribution alone was insufficient to account for the observed microstructural transformation [33]. Oscillatory acoustic streaming was proposed to repeatedly load dendrites within the mushy zone. The resulting fragments were considered to compete with the advancing columnar front after transport through the melt pool. Whether this sequence is maintained throughout the build depends on the stand-off distance, incidence angle, and the overlap between the acoustic footprint and the solidification region [68,69].

### 4.3. Texture Evolution and Orientation Dispersion

Crystallographic texture is a major source of mechanical anisotropy in additively manufactured metals. Directional heat flow and repeated epitaxial growth produce strong and spatially heterogeneous preferred orientations. As a result, the elastic response, yielding behavior, plastic deformation, and fracture resistance depend on the build orientation and loading direction [7,30]. Here, [uvw] denotes a crystallographic direction, ⟨uvw⟩ is a family of symmetry-equivalent directions, (hkl) presents a crystallographic plane, and {hkl} is a family of equivalent planes. The inverse pole figure (IPF) maps color individual grains according to their orientation relative to a specified sample direction. The pole figures display crystallographic plane normals or directions in the sample reference frame. Texture intensity is commonly expressed in multiples of a uniform distribution (MUD). A value of 1 represents a random orientation distribution, whereas a larger maximum MUD indicates stronger texture. These metrics distinguish crystallographic orientation inheritance from grain-size evolution.

The contact ultrasound results in Figure 11a distinguish changes in grain morphology from changes in crystallographic orientation. The NiTi B2 phase initially exhibited a strong cube texture, {001}⟨100⟩, with a maximum MUD of 18.00 [50]. At the intermediate ultrasonic level, the maximum MUD changed only slightly to 17.32, and the mean grain aspect ratio remained 3.6. Columnar growth and its associated preferred orientation therefore remained dominant. Under the highest ultrasonic intensity, the aspect ratio fell to 1.8, and the maximum MUD decreased to 3.48. The {100}, {110}, and {111} pole distributions became substantially more dispersed, indicating that the initial cube texture no longer dominated the microstructure. The simultaneous reduction in grain elongation and texture intensity is consistent with cavitation-associated nucleation and dendrite fragmentation, followed by acoustic-streaming-driven transport of viable fragments.

The interpretation of crystallographic orientation metrics depends on the crystal structure and phase constitution. In Ti6Al4V, ⟨001⟩ represents the preferred growth-direction family of cubic prior-β grains, whereas {0001} denotes the basal-plane family of the hexagonal α phase [30]. Ultrasonic excitation can interrupt prior-β epitaxial growth and broaden the β orientation distribution. The final α or α′ texture remains governed by the β → α transformation relationship and the subsequent cooling history. Consequently, reconstructed prior-β CET and weakened room-temperature α texture represent related but distinct microstructural outcomes.

Contact ultrasound studies have also reported reduced texture intensity in ER321 stainless steel [95], suppression of the strong {0001} texture in Mg-Al alloy [81], and reduced crystallographic anisotropy in laser-DED 17-4 PH stainless steel [97]. For cubic steels and nickel-base alloys, changes in the intensity and dispersion of the {100}, {110}, and {111} pole populations provide more meaningful evidence than a simple textured-random classification. Non-contact ultrasound weakens crystallographic texture by disrupting orientation inheritance through acoustic-streaming-induced disturbance of the solidification front. Zhang et al. [34] further showed that non-contact ultrasound reduced the maximum orientation density of 316L stainless steel from 9.384 to 7.505 (Figure 11b). For 316L stainless steel, the specimen fabricated without ultrasound exhibited a pronounced {100} texture with a maximum MUD of 16.57 [68]. The maximum MUD decreased to 4.86 at an incidence angle of 30° and to 8.13 at 150°. The corresponding {110} and {111} pole populations became progressively more dispersed. These observations indicate continuous texture weakening rather than complete randomization. Acoustic streaming does not rotate solid grains. Instead, it is considered to repeatedly load dendrites in the mushy zone, transport detached fragments, and increase the number of differently oriented growth units competing with the inherited {100} growth front. IPF maps should always be interpreted with reference to the sample direction specified in the original study. In the absence of this information, only orientation clustering and dispersion can be evaluated.

Grain refinement [26], geometric equiaxialization [50], CET [50], and texture weakening [68] remain distinct microstructural outcomes. A reduction in grain size does not necessarily eliminate a strong preferred orientation, and shortened columnar grains may still retain part of their epitaxial inheritance. Confirmation of CET requires evidence for the loss of trans-layer columnar continuity in addition to a reduced grain aspect ratio or lower texture intensity. The route-specific mechanisms discussed earlier are considered to increase the number of competing crystallographic orientations by contributing to dendrite fragmentation and fragment transport rather than by rotating solid grains. Texture weakening reduces the crystallographic contribution to mechanical anisotropy. The overall mechanical response remains influenced by the phase constitution, defect population, and loading direction [30,50,68].

### 4.4. Elemental Segregation and Second-Phase Distribution

Rapid solidification restricts solute diffusion and leaves enrichment along cells, dendrites, and grain boundaries. Repeated thermal exposure and phase transformation introduce additional phase and microstructural evolution in titanium alloys [98,99], aluminum matrix composites [25,100], steels [101], and other multiphase alloy systems [102]. Ultrasound modifies this state before complete solidification by enhancing liquid transport along the melt-pool tail and the solidification front. Under cavitation-active contact conditions, bubble collapse can generate pressure pulses and microjets that act with bulk acoustic streaming. Non-contact systems generate weaker streaming that acts together with Marangoni convection. Both mechanisms operate within the liquid and mushy zones and promote redistribution of solute and suspended phases.

Ultrasound shortened continuous solute-enrichment paths and inter-dendritic networks. It also produced finer and more dispersed secondary constituents. Strong contact excitation promoted local breakup and mixing [51,103]. Weaker gas-phase streaming improved liquid feeding and solute exchange within a footprint-defined region near the melt-pool tail [69].

Figure 12 presents representative evidence. Su et al. [51] utilized a contact ultrasound system to regulate alloying element segregation. They indicated that Cu enrichment along cellular structures and dendritic boundaries became less continuous. The mapped distributions of Al, Cu, Mg, and Zn also became more uniform across the observed fields (Figure 12a) [51]. A co-moving ultrasonic probe in AA7075-TiB_2_ WAAM reduced nano TiB_2_ particle agglomeration. It also lowered porosity and refined the solidification structure [104]. Niu et al. [66] showed that moving-contact ultrasound modified the elemental distribution in TiCp/Ti6Al4V composite coatings (Figure 12b). Without ultrasound, C was concentrated in relatively large TiC-rich regions. Ultrasonic assistance reduced the size and continuity of these localized C-rich regions and produced a finer and more dispersed C distribution. The Ti, Al, and V distributions remained broadly continuous within the matrix, indicating that the principal change involved the dissolution and redistribution of TiC-related C-rich constituents rather than complete homogenization of all elements. Ultrasonic laser AM of an Inconel 718/Ti6Al4V composite further reduced Ni segregation and suppressed Ti_2_Ni formation [67]. In Inconel 718, elongated Nb- and Mo-rich Laves constituents became finer and more dispersed, with less concentrated Nb enrichment [22,63]. In ultrasonic laser cladding of Inconel 718, the Laves-phase volume fraction decreased by 47.1%, and its morphology changed from elongated strips to fine granules [103]. Under non-contact ultrasound, B and C enrichment in cellular or inter-dendritic regions decreased [69]. The mapped distributions of B, C, Cr, and Fe also became more uniform. The influence of stand-off distance depended on the overlap between the local streaming field and the melt-pool tail.

Enhanced melt transport links ultrasound with reduced segregation across these alloy systems [51,69,103]. The mapped fields indicate local solute redistribution instead of complete homogenization. In the cited contact studies, rapid local redistribution was attributed to cavitation-associated effects and acoustic streaming [51,103]. Non-contact excitation relied mainly on weaker streaming-driven solute exchange [69]. Shorter solute-rich boundary networks and finer secondary phases reduced preferred crack-initiation sites. These changes contributed to the strength–ductility response with grain refinement and defect suppression [24,51].

Across defects, grains, texture, and solute distribution, material evolution follows the energy delivered to the active liquid and mushy zones. Contact ultrasound can provide stronger acoustic forcing and, when the melt-side threshold is reached, cavitation-associated effects together with acoustic streaming. Excessive or unstable coupling may damage track integrity [23,24,49]. Non-contact excitation preserves toolpath accessibility and depends on accurate placement of the acoustic footprint to maintain streaming near the melt-pool tail [34,68,69]. The limits of each delivery route determine process stability. Non-contact systems remain sensitive to insufficient overlap between the acoustic field and the melt pool [33,34,68,69,70].

## 5. Multilayer Build Quality and Mechanical-Property Response

### 5.1. Multilayer Build Quality

Mechanical properties become meaningful only after the process produces a testable specimen [7]. A local reduction in porosity or grain size provides limited engineering value if the same condition also causes discontinuous tracks, severe wall waviness, collapse, notches, or unstable overlap. Geometry and internal quality jointly determine structural performance. Surface notches influence tensile and fatigue behavior. Porosity [69], sampling direction [50], machining allowance [50], and test history [7] may mask the contribution of microstructure. Therefore, reliable build quality is a prerequisite for attributing property changes to ultrasonic melt-pool control [31,32,60,65].

Printability can be assessed at three evidence levels: track-scale stability, regular multilayer formation, and component-scale adaptability. Track-scale evidence includes continuous deposition, repeatable track width and height, stable surface flow, and the absence of local collapse. Regular multilayer builds require consistent overlap, continuous walls, and retained geometry throughout the build height. Component-scale evidence further includes curved toolpaths, variable cross-sections, tall or long builds, and changing access conditions. Figure 13 mainly presents first- and second-level evidence. Peng et al. [60] demonstrated continuous multilayer deposition of Inconel 718 with platform-based contact ultrasound (Figure 13a). The ultrasonic response was more pronounced in the lower part of the wall and weakened toward the upper region, implying that the effective vibration transmitted through the substrate decreased with increasing build height. This height-dependent response limits the uniformity of ultrasonic input during multilayer buildup. High-intensity contact ultrasound also modified surface ripples and interlayer waviness [65]. Hamilton et al. [32] applied moving-contact ultrasound-assisted wire-laser DED (LW-DED) (Figure 13b) to produce an approximately 85 mm-long specimen. A 12-layer thin wall with a height of about 14.5–15 mm was fabricated under selected conditions [32]. These results demonstrate that ultrasonic assistance can be maintained during continuous track deposition and regular multilayer buildup.

Non-contact ultrasound studies continue to emphasize continuous tracks and regular walls or blocks. This approach improved powder spreading, interlayer continuity, and surface regularity (Figure 13c) [34]. Representative non-contact studies produced taller and more regular specimens [33,68] without transmitting vibration through the growing component. Compared with the ultrasound-free condition, low-intensity non-contact ultrasound maintained regular high-wall formation, whereas high-intensity contact ultrasound strongly disturbed the lower deposited layers and prevented stable wall formation [33]. The ultrasonic source moves with the deposition head. This configuration avoids the conflict between low-layer and high-layer energy transmission that exists in platform excitation. Performance still depends on appropriate stand-off distance, incidence angle, shielding-gas flow, and acoustic-field coverage relative to the melt pool. However, curved toolpaths, variable cross-sections, and component-scale builds have received limited investigation.

Current studies have demonstrated continuous-track formation [32,34,60] and the feasibility of regular multilayer deposition [33,62,65]. These results support specimen extraction and mechanical testing within individual studies [32,33,34]. Stable deposition under changing geometry and access conditions has yet to be demonstrated at component scale. Mechanical property comparisons become more reliable when ultrasonic and reference specimens share comparable geometry and defect content, consistent sampling directions, and matched surface and test conditions [50,69].

Each ultrasonic implementation faces a distinct limitation at the component scale. Platform systems are affected by height-dependent energy input and evolving structural vibration modes [30,50,65]. Moving-contact systems require stable acoustic coupling over changing surfaces while avoiding damage or contamination of the deposited material [32,62,66]. Non-contact systems require stable alignment between the acoustic footprint and a moving, inclined, or curved melt pool [33,34,68]. Sufficient acoustic energy transfer through the shielding gas is also essential [33,69,70]. Therefore, the experimental validation under these conditions remains limited [32,33,34,62,68].

### 5.2. Mechanical-Property Responses of Ultrasound-Assisted Metal AM

Hardness, yield strength (YS), ultimate tensile strength (UTS), and elongation reflect the overall condition of the printed material. Current evidence covers contact ultrasound applied to nickel-base alloys [60,105,106,107], steels [108,109], titanium alloys [110,111,112,113], NiTi alloys [50], aluminum alloys [51], and particle-reinforced composites [104,112]. A smaller number of non-contact ultrasound studies have also been reported [34,68,69,70]. Within each study, the measured properties result from the combined effects of defects, grain structure, texture, phase constitution, residual stress, and geometry. Contact ultrasound results are generally interpreted in terms of cavitation- and acoustic-streaming-induced melt-pool mixing. Non-contact ultrasound results are commonly explained by the interaction between acoustic streaming and Marangoni flow near the melt-pool tail. Differences in material, build orientation, specimen geometry, and testing method make within-study comparisons the most appropriate.

Table 2 summarizes 21 within-study mechanical-property comparisons from five contact studies [23,24,44,51,89] and five non-contact ultrasound studies [33,34,68,69,70]. The contact results cover Inconel 718, AlMgScZr, GH5188, AA7075, and AZ31. The reported responses include hardness, yield strength, ultimate tensile strength, and elongation. The non-contact ultrasound results cover Inconel 718 and 316L stainless steel, together with Fe-based deposits on 42CrMoA steel. Each row compares ultrasound and no-ultrasound conditions within the same study; testing direction and source-side or melt-pool parameters are retained when the source reports them.

Figure 14 compares representative tensile results from contact-assisted and gas-phase non-contact systems. Zhang et al. [50] presented a NiTi dataset including transverse-direction (TD) and build-direction (BD) specimens without ultrasound and under two contact-ultrasound conditions (Figure 14a). The smaller difference between the two directions agrees with the measured CET and reduced texture concentration. However, directional dependence remained. Gas-phase non-contact ultrasound increased the ultimate tensile strength of 316L from 691 to 707 MPa (~2.3%) (Figure 14b) [34]. The elongations were similar, implying that the modest strength increase was achieved without an evident loss of ductility. The Fe-based gas-phase non-contact results showed a non-monotonic response to stand-off distance [69]. The highest ultimate tensile strength reached 1281.3 MPa at a stand-off distance of 80 mm. This value was 30.1% higher than that of the no-ultrasound counterpart. They present the potential of an ultrasound-assisted strategy for simultaneous strengthening.

## 6. Conclusions and Perspectives

1. Energy delivery determines the melt-pool response. Platform-based contact provides strong acoustic coupling through the solid substrate. The delivered energy at the active melt pool changes with build height, damping, reflection, resonance, clamping conditions, and structural vibration modes. Moving-contact shortens the transmission path and reduces height dependence. It also requires stable local coupling, precise synchronization, wear resistance, and adequate process access. Non-contact excitation follows changing build geometry without transmitting vibration through the component. Acoustic attenuation in the gas and the large gas–liquid impedance mismatch substantially reduce energy transfer. These conclusions apply to the investigated fusion-based AM systems, particularly DED/LDED and WAAM, and should not be generalized to all metal AM processes, including LPBF.
2. Delivery route determines the dominant physical mechanism. Strong contact coupling can produce cavitation-associated effects when the melt-side pressure reaches the required threshold, together with acoustic streaming, periodic pressure loading, and inertial melt motion. Measurements reported for non-contact systems indicate acoustic intensities approximately one order of magnitude below the cavitation threshold for metallic melts. Their dominant responses are therefore interpreted as acoustic streaming, cyclic pressure, and coupling with Marangoni convection. Experimental observations of melt flow and final microstructure, together with numerical predictions, support a model-based interpretation involving oscillatory melt flow, repeated dendrite loading, possible fatigue fragmentation, fragment transport, and interrupted columnar growth in the mushy zone.
3. Delivered acoustic energy governs defect evolution and microstructural development. Within the investigated systems, contact ultrasound was associated with pore suppression, grain refinement, CET, texture weakening, solute redistribution, and second-phase dispersion. Excessive input or inconsistent energy delivery along the build height also reduced process stability. Non-contact excitation produced weaker and more localized effects. Accurate alignment of the acoustic footprint improved bubble escape, grain morphology, texture dispersion, and compositional uniformity. Grain refinement, equiaxed grain formation, CET, and texture weakening should be regarded as distinct microstructural responses rather than interchangeable descriptions.
4. Build-scale evidence includes track continuity, multilayer stability, and component-scale adaptability. Both contact and non-contact ultrasound approaches have achieved continuous tracks and regular walls or blocks. Platform excitation remains constrained by height-dependent energy transmission, while moving-contact methods rely on stable interface control and hardware integration. Non-contact excitation requires accurate acoustic-field positioning and efficient energy coupling. Evidence for curved toolpaths, variable cross sections, and full-scale component fabrication remains limited. For engineering implementation, stable and repeatable fabrication should be established before property optimization. Mechanical property variations result from the combined effects of ultrasonic melt-pool regulation on defects, grain structure, texture, solute distribution, second phases, and residual stress. Within the investigated DED/LDED and WAAM studies, hardness and strength often increased under selected ultrasonic conditions, whereas elongation responses depended on the alloy system and processing conditions.
5. Future work should combine direct measurements, or validated indirect measurements, of the acoustic energy delivered to the melt pool with height-resolved transmission mapping, gas–liquid transmission losses, in situ process monitoring, and closed-loop control of source position or acoustic output. Manufacturing validation should extend to tall structures, curved toolpaths, variable cross-section, and long-duration builds. Integrating acoustic measurement with manufacturing validation would allow each ultrasonic delivery route to maintain sufficient acoustic forcing and effective field overlap at the active melt pool.

## Figures and Tables

**Figure 1:**
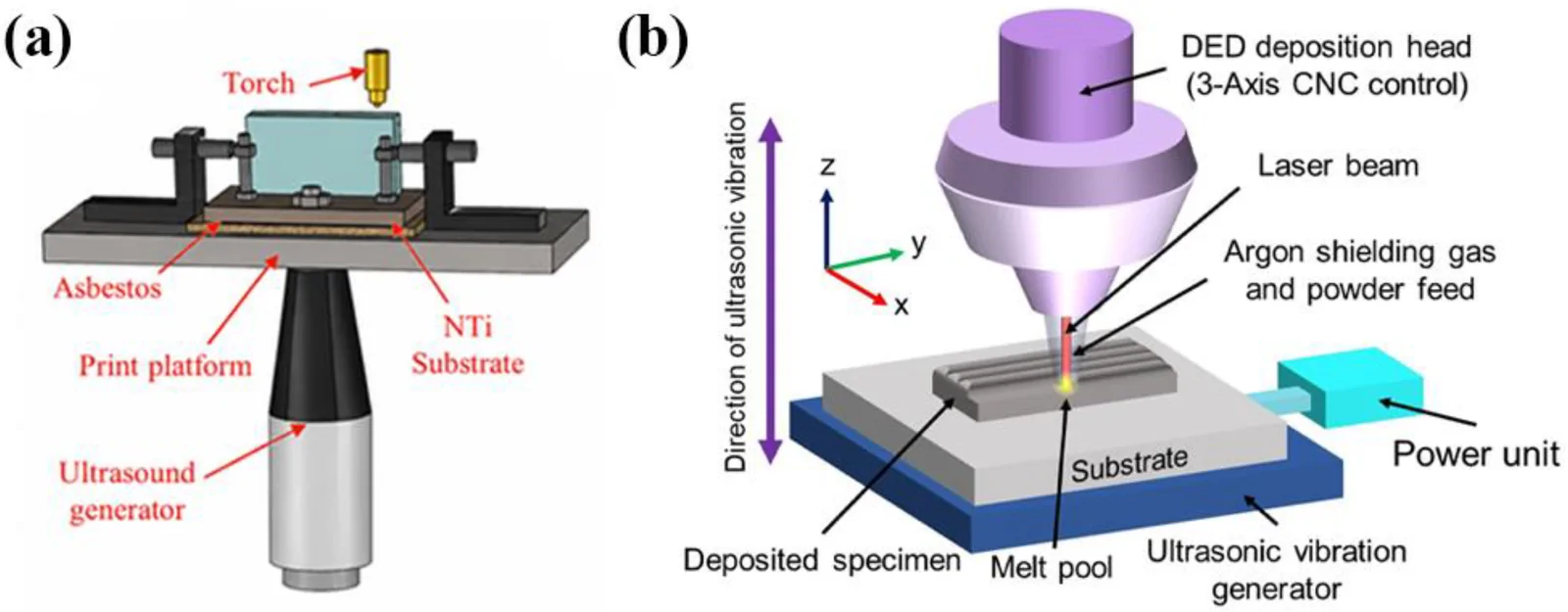
Illustration of the platform-based ultrasound-assisted AM equipment. (**a**) Ultrasound introduced through the build platform in wire-arc DED [50], (**b**) platform vibration-assisted LDED system and specimen layout [51].

**Figure 2:**
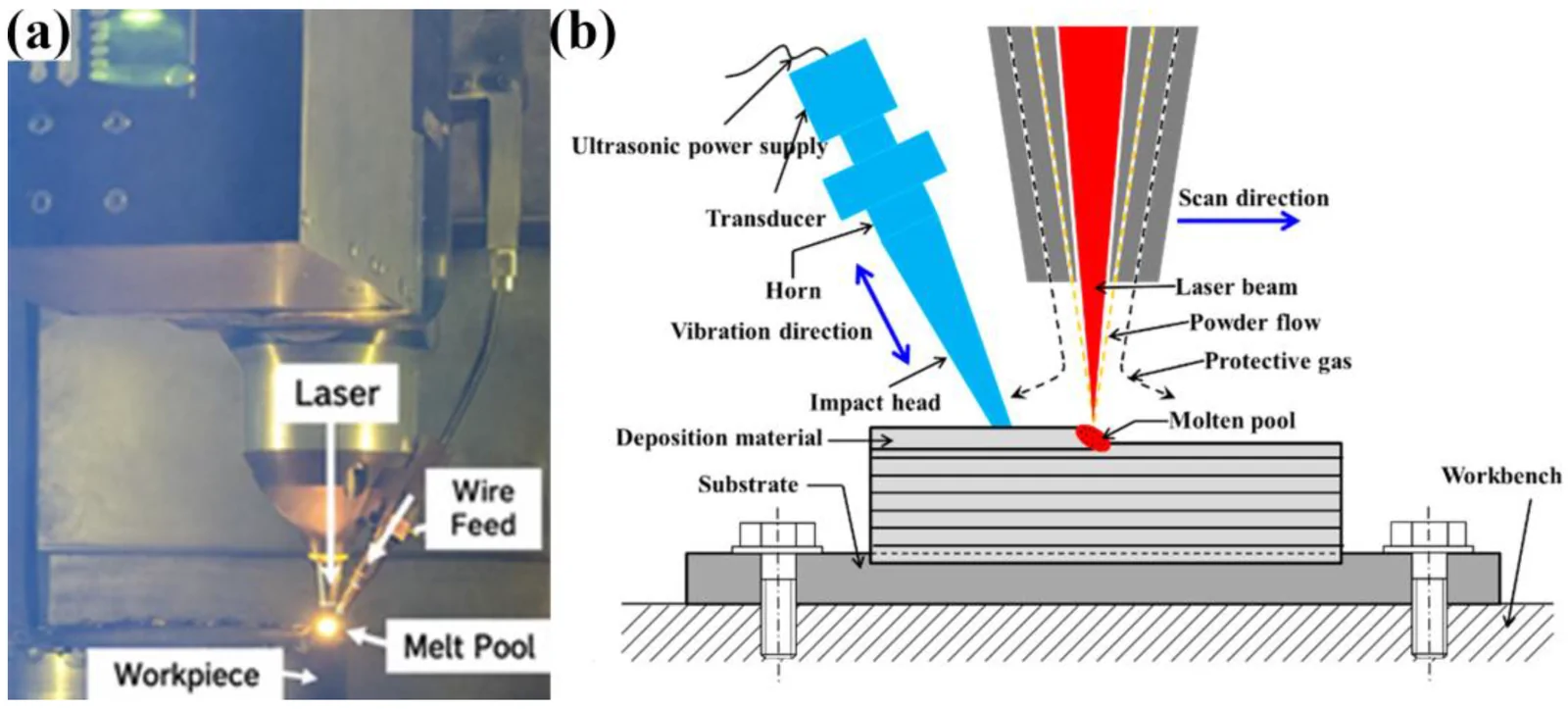
Moving-contact ultrasound near the heat source: (**a**) local vibration introduced in the processing region [32]; (**b**) follow-up ultrasound-assisted laser additive manufacturing configuration [66]. In panel (**b**), the blue arrows indicate the scan direction and ultrasonic-vibration direction. The colors are used to distinguish the schematic components which indicated by black arrows.

**Figure 3:**
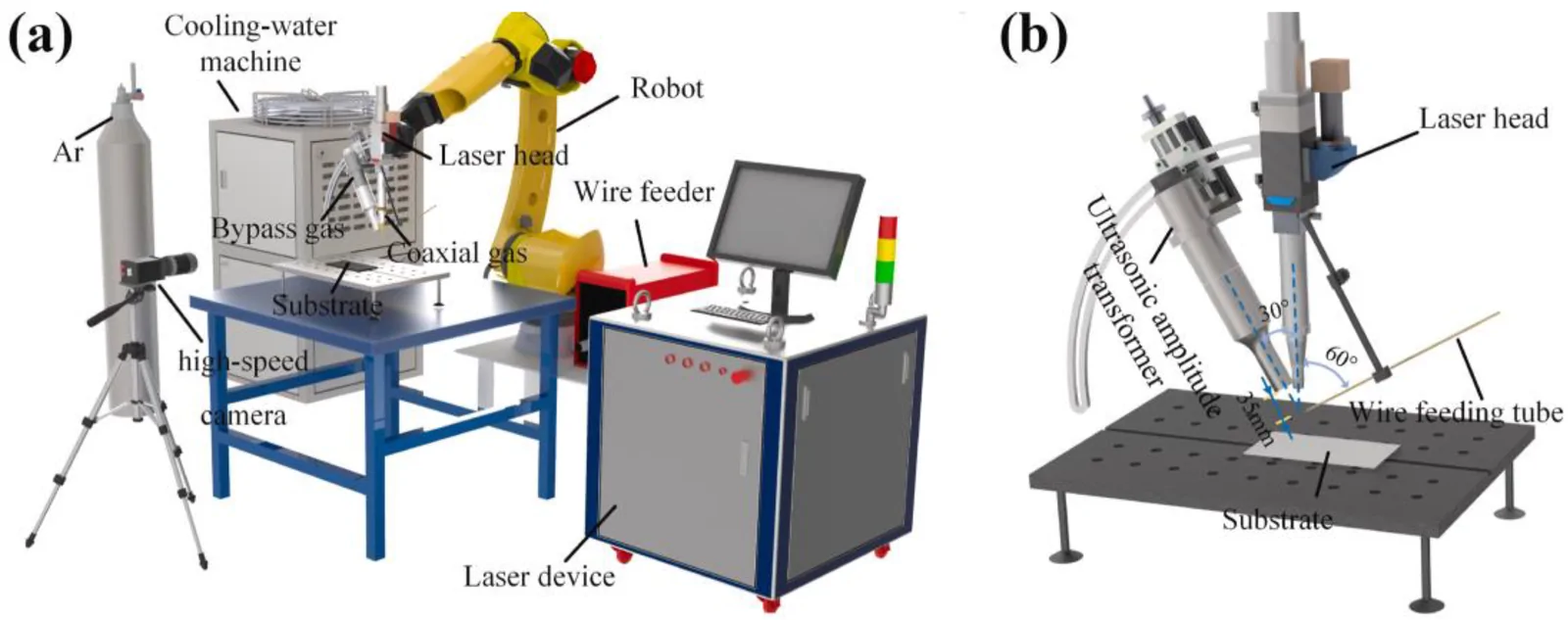
Non-contact ultrasound-assisted AM system [34]: (**a**) external acoustic-source arrangement in wire-fed LDED; (**b**) ultrasound-assisted deposition device.

**Figure 4:**
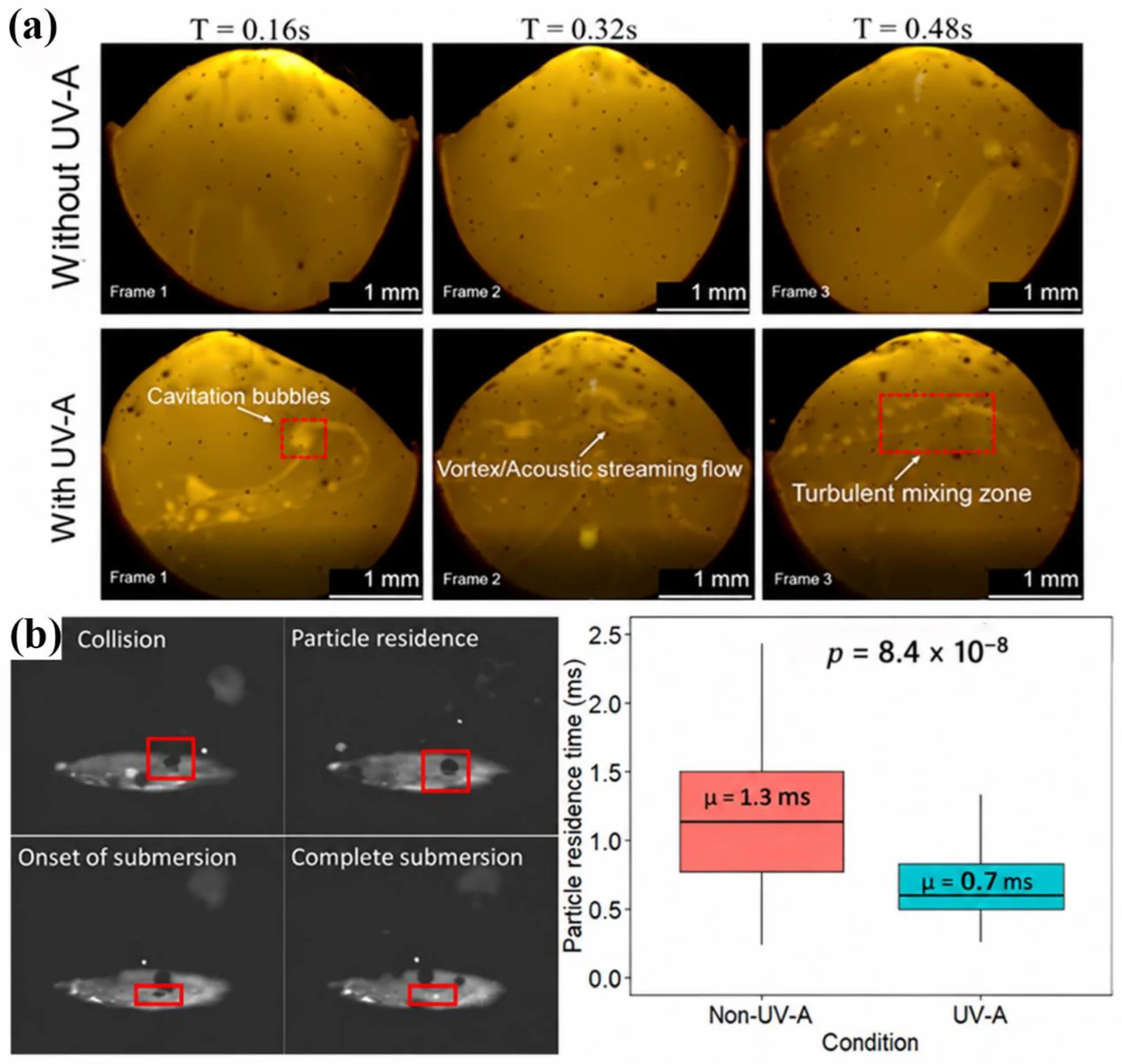
Melt-pool flow and transport response under contact ultrasound-assisted system: (**a**) in situ high-speed images of melt-pool flow with and without contact ultrasound [75]; (**b**) particle immersion sequence and residence-time distribution [49].

**Figure 5:**
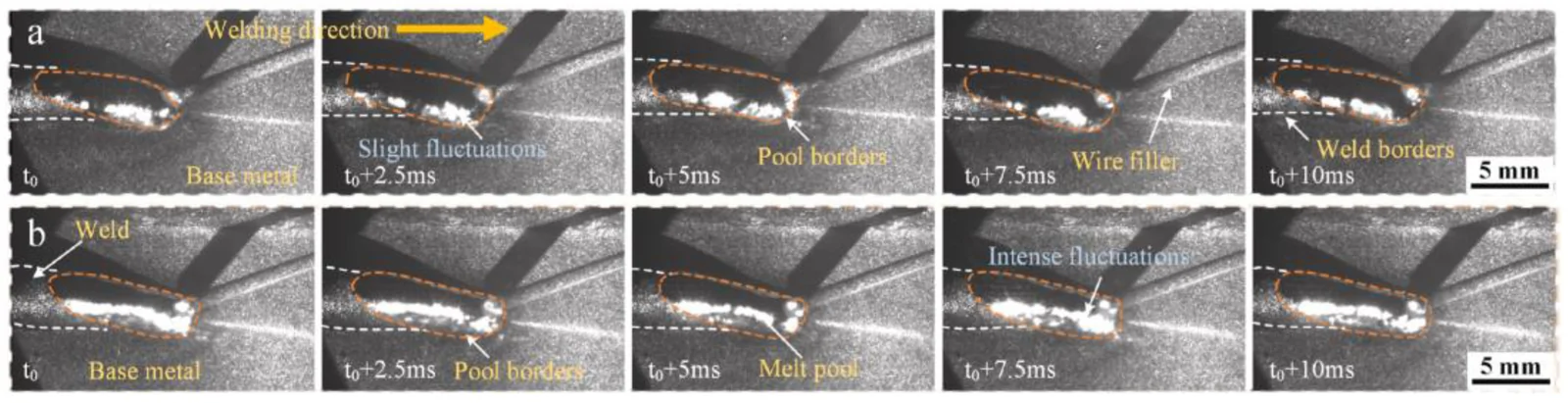
Time-resolved melt-pool morphologies during conventional and non-contact ultrasound-assisted LDED of 316L stainless steel: (**a**) LDED; (**b**) non-contact ultrasound-assisted LDED [34].

**Figure 6:**
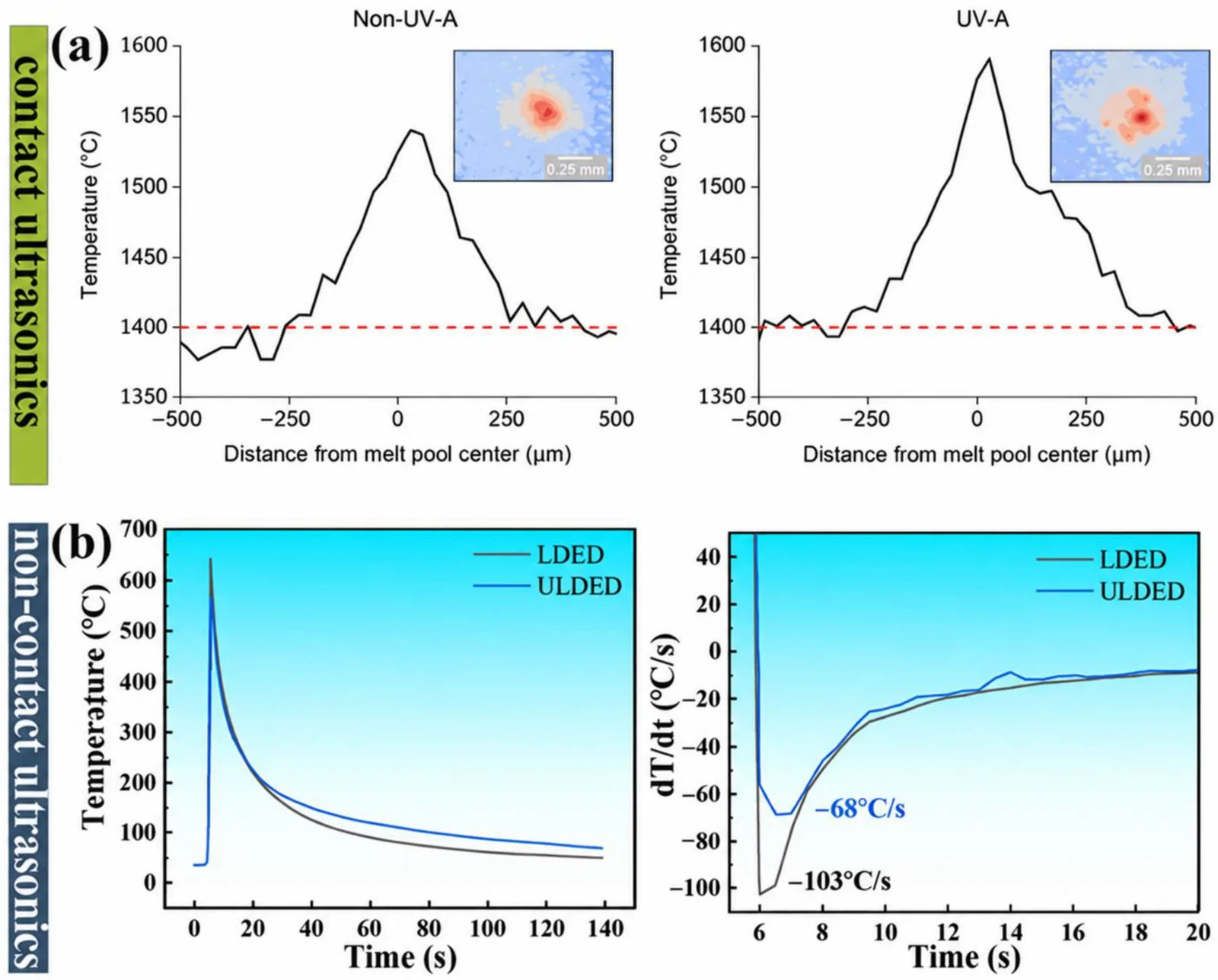
Representative thermal responses of the melt pool under contact and non-contact ultrasound-assisted LDED: (**a**) melt-pool temperature profiles and thermal maps without and with contact ultrasound [49]; (**b**) thermal cycles and cooling-rate histories without and with non-contact ultrasound [34].

**Figure 7:**
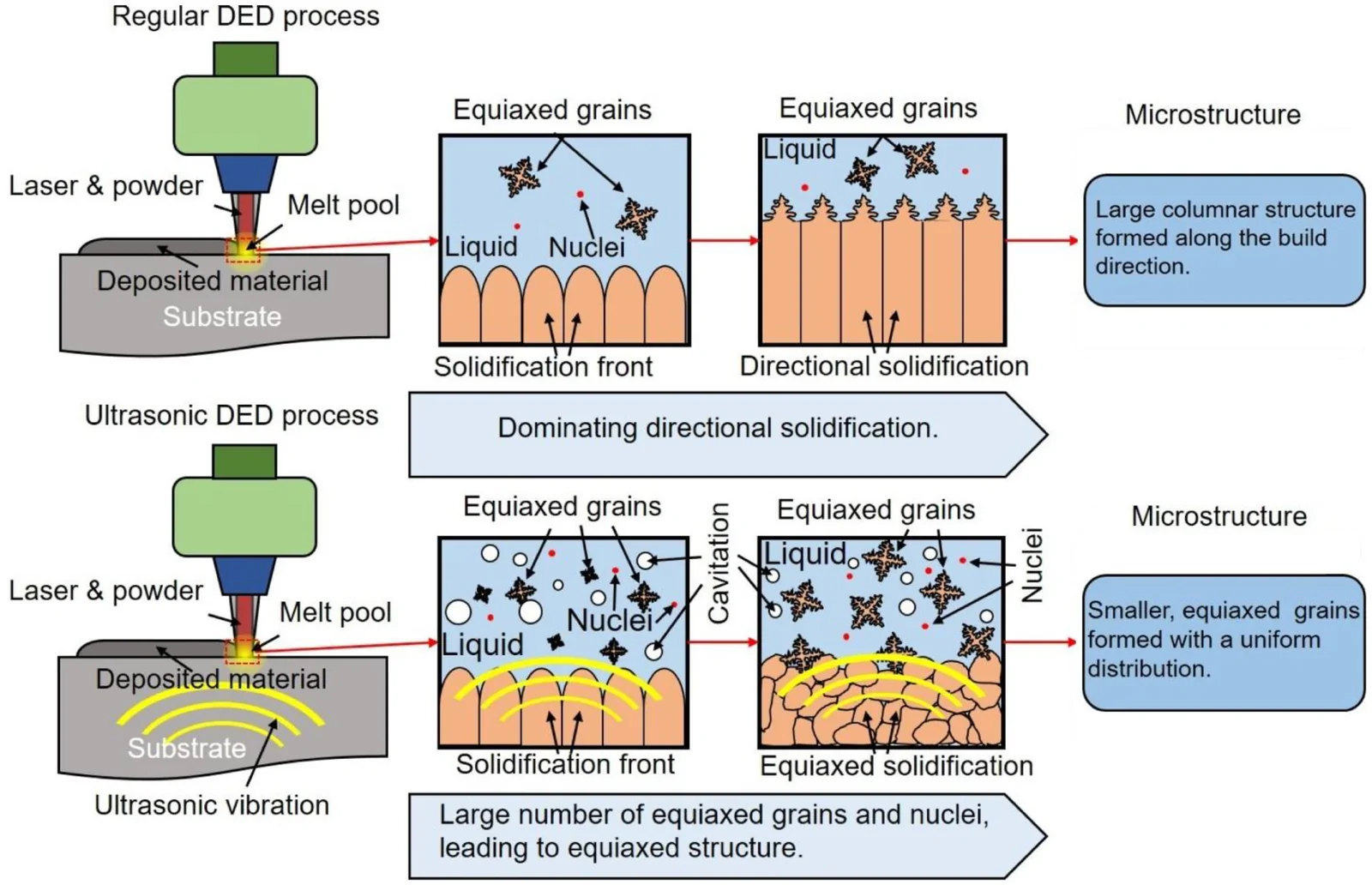
Schematic of cavitation- and acoustic-streaming-assisted equiaxed-grain formation under contact ultrasound [51].

**Figure 8:**
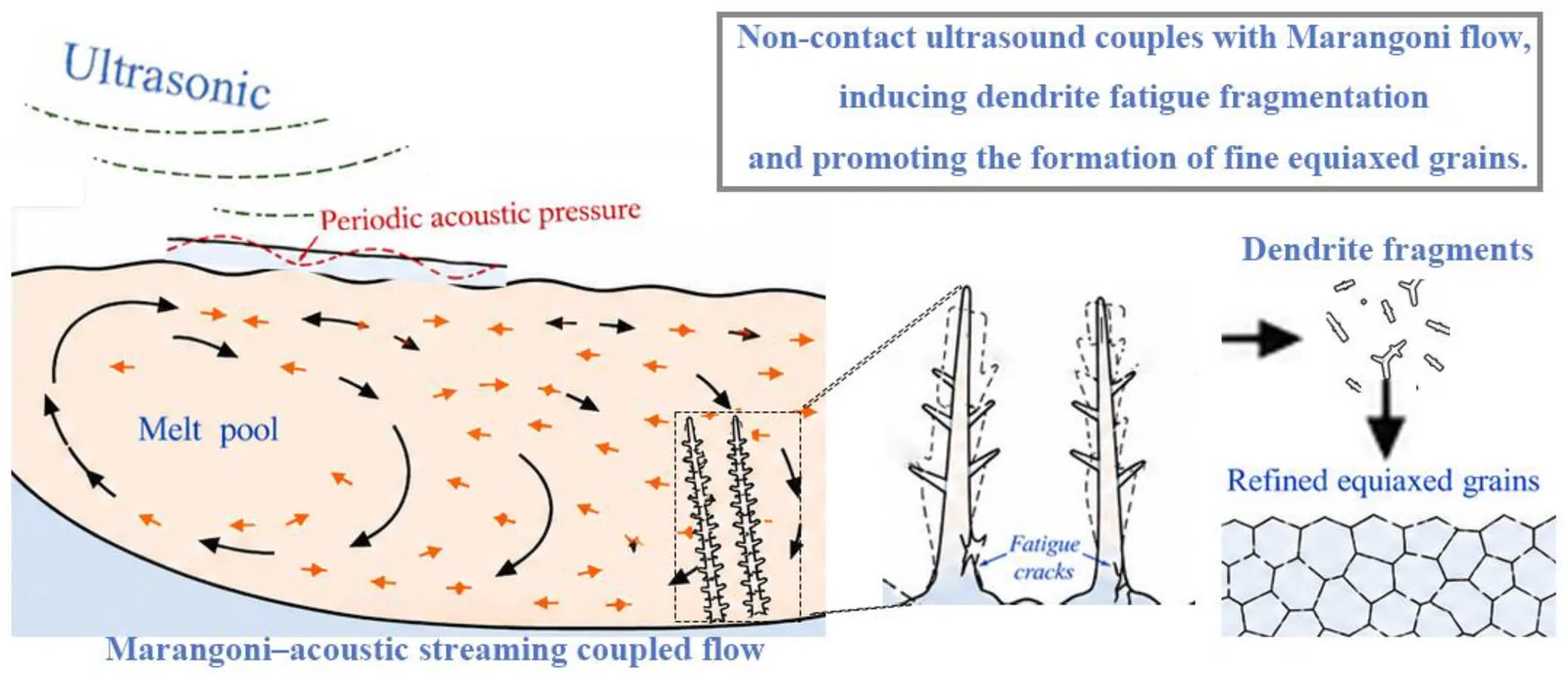
Schematic of Marangoni–acoustic-streaming coupled flow, dendrite fatigue fragmentation, and equiaxed-grain formation under non-contact ultrasound. The black arrows indicate Marangoni flow. The orange arrows show ultra-sound induced acoustic streaming.

**Figure 9:**
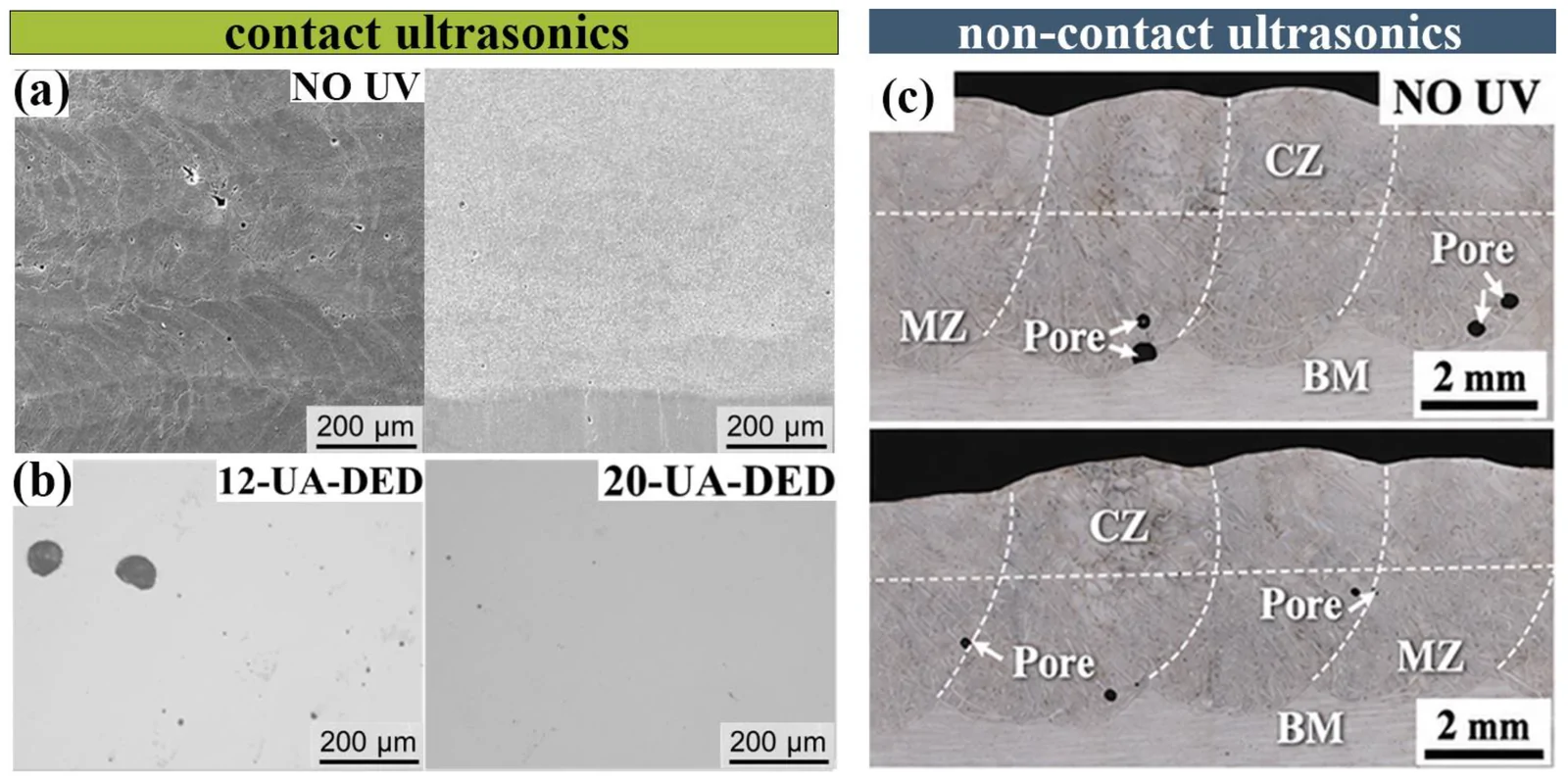
Internal-defect responses under contact and non-contact ultrasound-assisted systems: (**a**) pores and shrinkage cavities in the deposits fabricated with and without contact ultrasound [51]; (**b**) pore morphologies with ultrasonic frequencies of 12 and 20 kHz [60]; (**c**) cross-sectional pore morphologies with and without non-contact ultrasound in 316L stainless steel [34].

**Figure 10:**
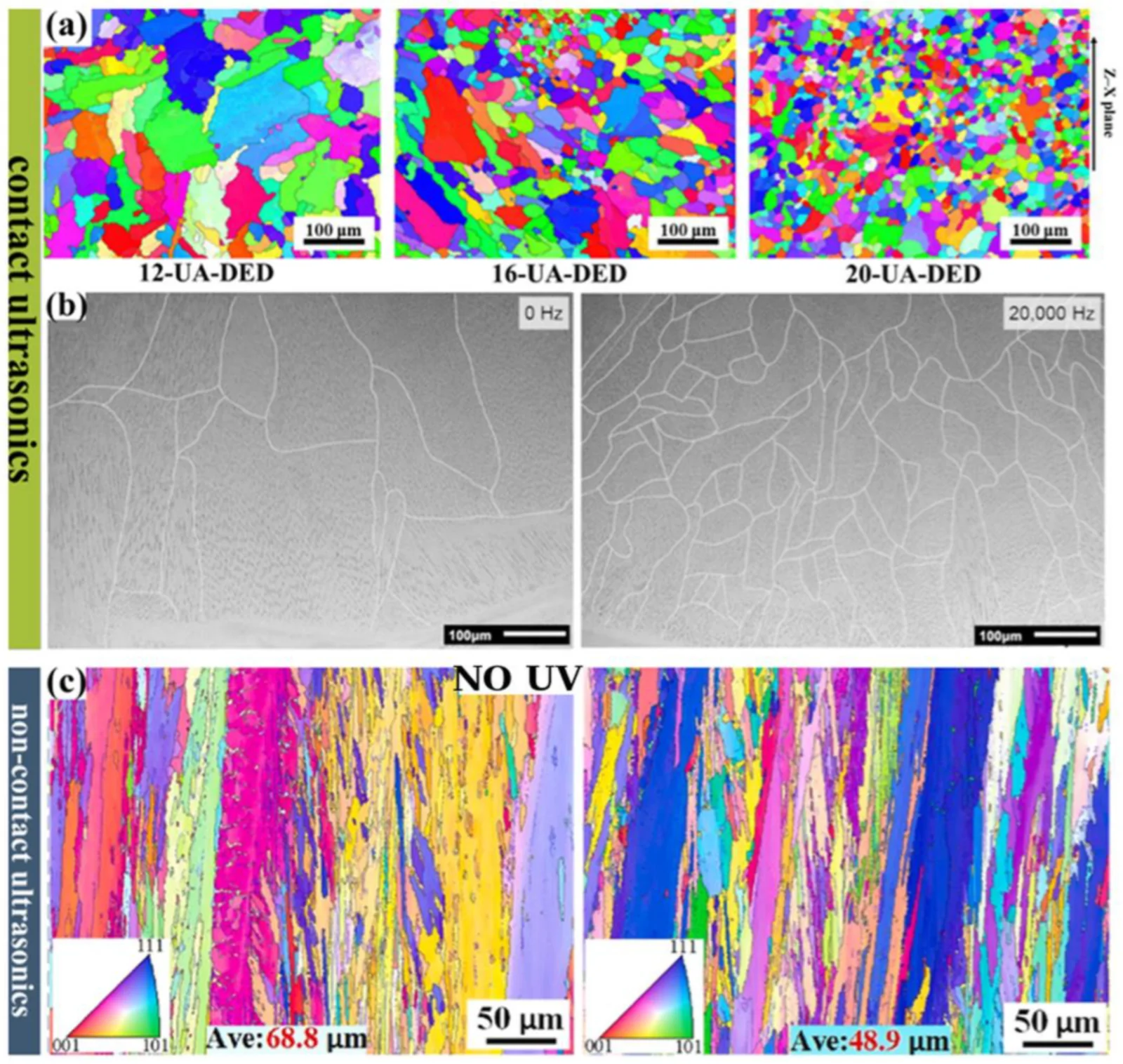
Grain-scale and local microstructural evolution with contact and non-contact ultrasound-assisted systems: (**a**) EBSD inverse pole figure maps of Inconel 718 deposited at ultrasonic frequencies of 12, 16, and 20 kHz [60]; (**b**) microstructures with and without moving-contact ultrasound [32]; (**c**) EBSD inverse pole figure maps of 316L stainless steel with and without non-contact ultrasound [34].

**Figure 11:**
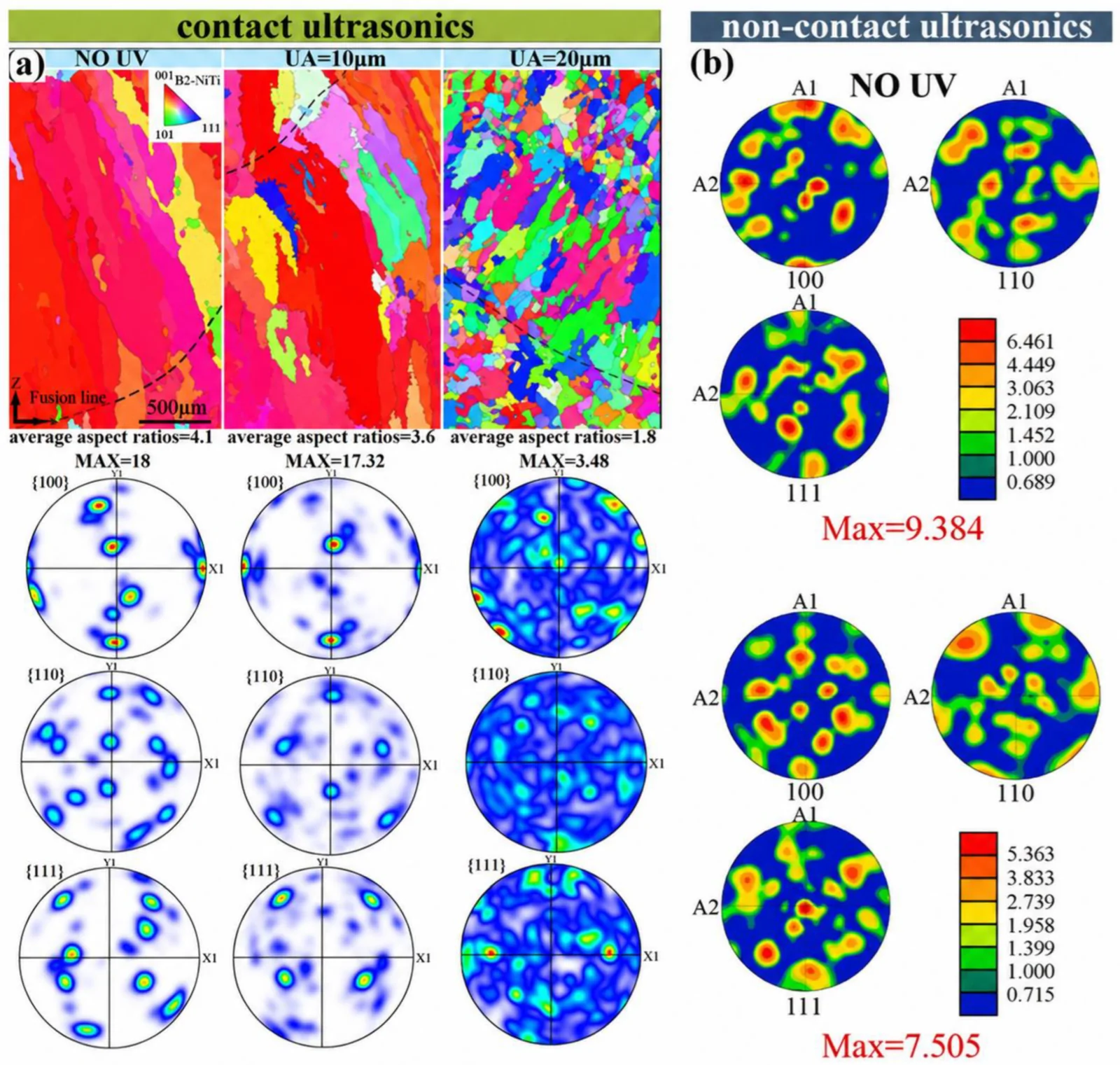
Grain morphology, columnar-to-equiaxed transition, and texture evolution induced by contact and non-contact ultrasound: (**a**) NiTi grain morphology, aspect ratio, and pole figures with contact ultrasound [50]; (**b**) the pole figure maps of 316L stainless steel with and without non-contact ultrasound [34].

**Figure 12:**
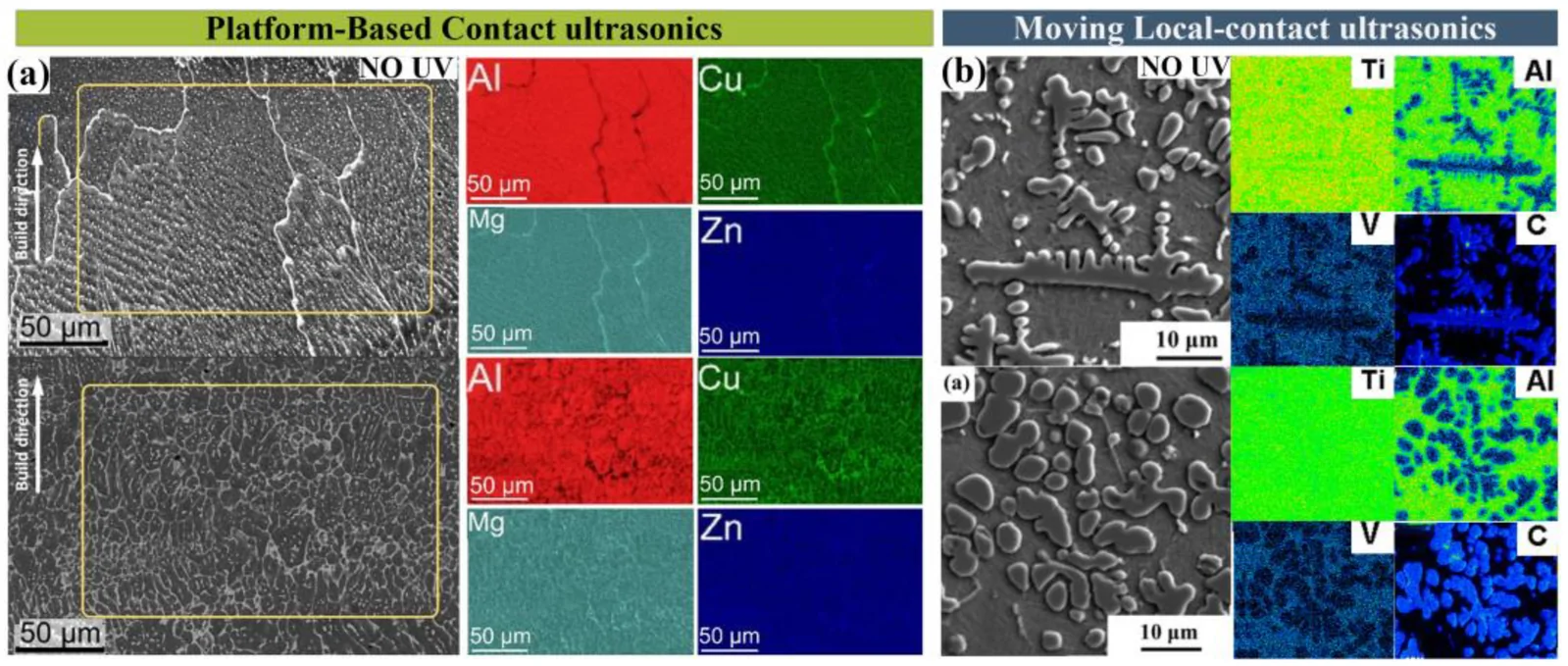
Solute redistribution and second-phase evolution in the alloys fabricated via U-FAAM: (**a**) the major alloying element distributions with and without contact ultrasound [51]; (**b**) microstructure and elemental distributions of TiCp/Ti6Al4V composite coatings without and with moving-contact ultrasound [66].

**Figure 13:**
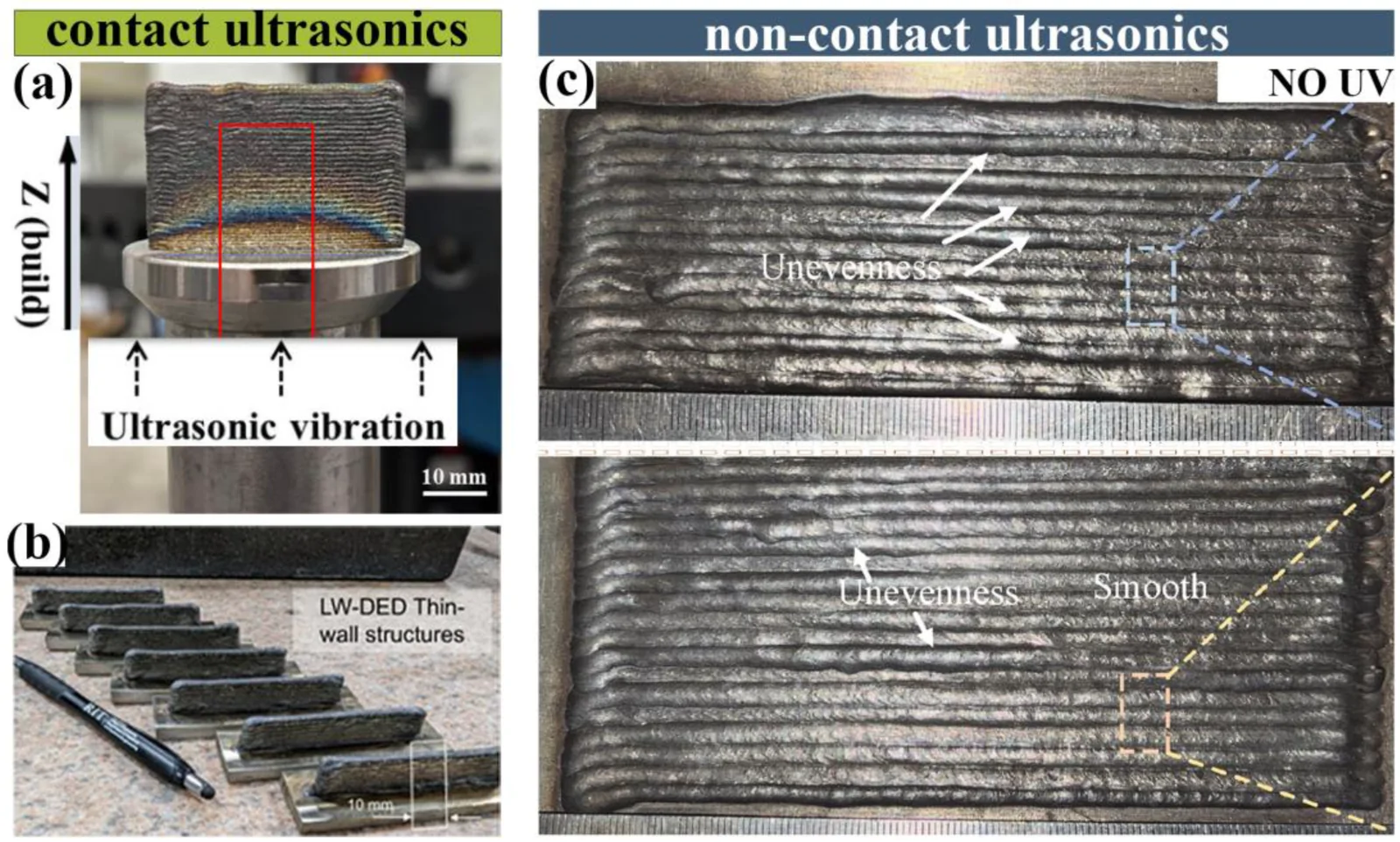
Multilayer build quality under contact and non-contact ultrasound: (**a**) surface morphology of multilayer Inconel 718 builds deposited with various ultrasonic frequencies [60]; (**b**) moving-contact ultrasound-assisted LW-DED specimen [32]; (**c**) wall surfaces with and without non-contact ultrasound [34].

**Figure 14:**
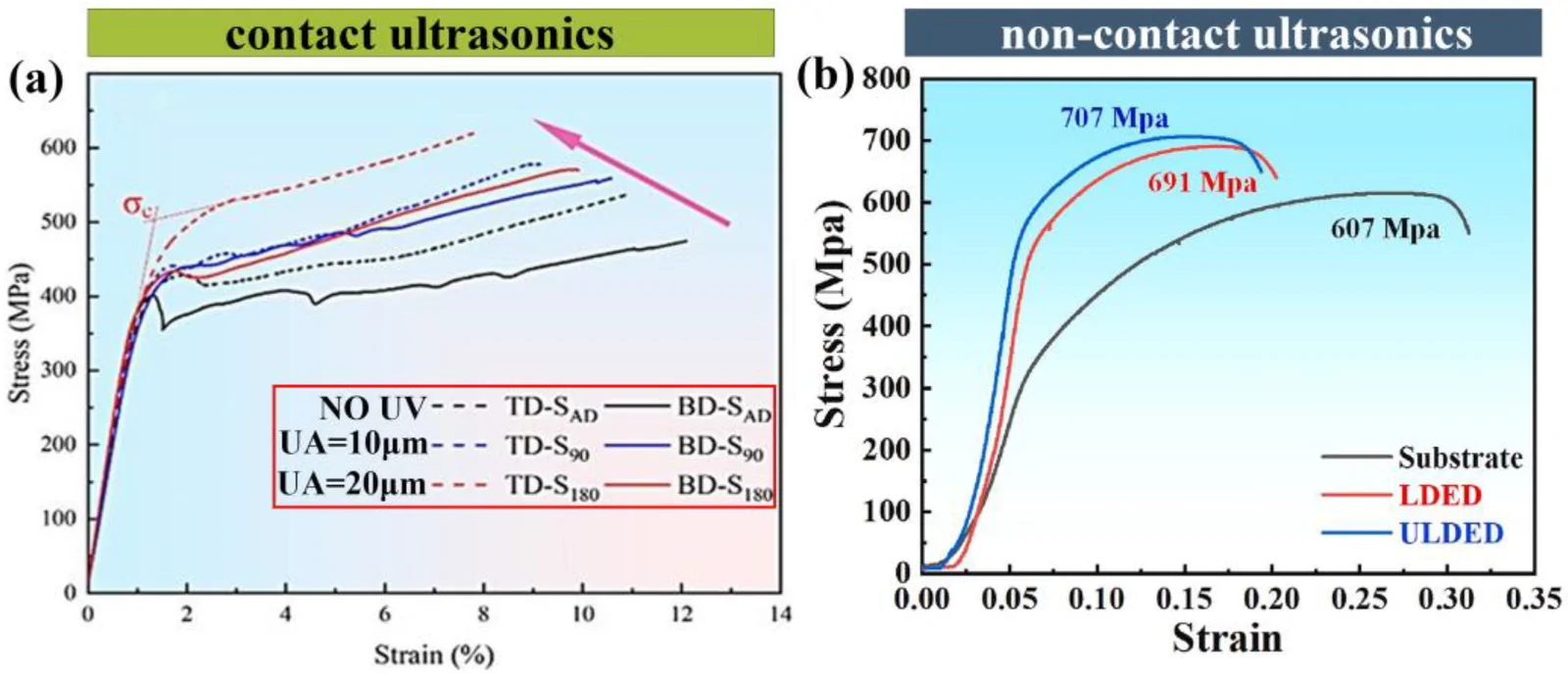
The representative tensile stress–strain curves with and without ultrasound assistance: (**a**) WAAM-ed NiTi with contact ultrasound [50]; (**b**) tensile stress–strain curves of 316L substrate, LDED, and non-contact ultrasound-assisted LDED specimens [34].

**Table 1:** Comparative characteristics of platform-based contact, moving-contact, and non-contact ultrasound input routes in U-FAAM.

Characteristic	Platform-Based Contact	Moving-Contact	Non-Contact
Acoustic-energy path	Solid-state transmission from the substrate or build platform through the growing component to the melt pool [30,49,50].	Short-range solid-state transmission through a local tool, adjacent solid/semisolid region, or vibrating feed wire [32,61,62].	Gas-phase transmission through air, argon, or shielding gas to the melt-pool surface or trailing solidification region [33,34,68].
Main factors affecting acoustic-energy delivery	Clamping conditions; solid-path attenuation and reflection; component structural modes [49,50,54,55].	Local coupling stability; tool–pool alignment; motion synchronization [32,62,66].	Stand-off distance; incidence angle; acoustic-field coverage; shielding-gas flow; free-surface geometry [33,34,68,69].
Dependence on build height	Greater dependence; the propagation path lengthens, and structural response evolves with build height [30,49,50,65].	Reduced dependence; the local transmission path remains short near the active melt pool [32,61,62].	Reduced dependence when source–pool geometry is maintained; no transmission through the growing component [33,34,68].
Compatibility with AM processes and component geometries	Demonstrated in DED/LDED/LMD, WAAM, and selected LPBF systems using platform excitation; better suited to regular component geometries [30,44,49,54,56].	Mainly applicable to open DED and WAAM systems; requires continuous tool access and synchronized motion [32,61,62,66].	Mainly demonstrated in laser DED/LMD systems; no rigid contact and greater flexibility for variable or complex component geometries [33,34,68,69,70].

**Table 2:** The representative within-study mechanical-property responses reported for contact and non-contact ultrasound-assisted metal AM.

AM Process	Input Route	Material	Ultrasonic Parameters	Mechanical Property	With Ultrasound	Without Ultrasound	Units	Change (%)	Ref.
LDED	Contact	Inconel 718	f = 41 kHz; A ≈ 5 μm	Hardness	280.6	236.1	HV1.0	+18.8	[23]
LDED	Contact	AlMgScZr	f = 20 kHz; A = 16.3 μm	YS	215	198	MPa	+8.6	[24]
				UTS	392	348	MPa	+12.6	
				Elongation	22.1	18.1	%	+22.1	
LPBF	Contact	GH5188	f = 40 kHz; P = 15 W; A ≈ 2.5 μm	Hardness	300.60	287.67	HV0.1	+4.5	[44]
				YS	731	699	MPa	+4.6	
				UTS	1032.67	1006.67	MPa	+2.6	
				Elongation	45.85	43.43	%	+5.6	
LMD	Contact	AA7075	f = 30 kHz; generator P = 250 W	Hardness	110.2	93.1	HV0.1	+18.4	[51]
				UTS	314.7	226.8	MPa	+38.8	
WA-DED	Contact	AZ31	generator P = 90 W; BD	YS	91	70	MPa	+30.0	[89]
				UTS	236	163	MPa	+44.8	
				Elongation	19.1	6.6	%	+189.4	
LDED	Non-contact	Inconel 718	f = 20 kHz; d = 20.6 mm; I = 17.5 W·cm^−2^	YS	582	456	MPa	+27.6	[33]
				UTS	994	915	MPa	+8.6	
				Elongation	40.1	41.5	%	−3.4	
LDED	Non-contact	316L	f = 20 kHz; d = 35 mm; horn Amax = 40 μm	UTS (transverse)	707	691	MPa	+2.3	[34]
LDED	Non-contact	316L	θ = 30°; generator setting = 100%	Hardness range	198–235	155–212	MPa	—	[68]
LMD	Non-contact	Fe-based deposit/42CrMoA	f = 20 kHz; d = 50 mm	UTS	1162.6	984.7	MPa	+18.1	[69]
			f = 20 kHz; d = 80 mm	UTS	1281.3	984.7	MPa	+30.1	
LMD	Non-contact	Fe-based deposit/42CrMoA	d = 80 mm; source A = 30 μm	Average hardness	772.9	677.1 *	HV	+14.1	[70]

Note: YS, yield strength; UTS, ultimate tensile strength; HV, Vickers hardness; f, frequency; A, amplitude; P, ultrasonic-generator power; d, source-to-pool stand-off distance; I, measured ultrasonic intensity in the melt pool; θ, incidence angle; BD, build direction. For single-valued comparisons, Change (%) = (value with ultrasound − value without ultrasound)/value without ultrasound × 100. The hardness ranges of 198–235 and 155–212 MPa [68] are retained exactly as given by the source; because the paper also describes Vickers hardness measurements, this is treated as a source-level reporting inconsistency rather than converted to HV without author confirmation. * The no-ultrasound hardness was back-calculated from the source-reported values 772.9 HV and a 95.8 HV increase: 772.9 − 95.8 = 677.1 HV [70]. Source-side generator settings are identified explicitly and should not be interpreted as the acoustic condition inside the melt pool.

## Data Availability

No new data were created or analyzed in this study. Data sharing is not applicable to this article.
